# Self‐reported sleep quality is more closely associated with mental and physical health than chronotype and sleep duration in young adults: A multi‐instrument analysis

**DOI:** 10.1111/jsr.13152

**Published:** 2020-08-11

**Authors:** Khyla Muzni, John A. Groeger, Derk‐Jan Dijk, Alpar S. Lazar

**Affiliations:** ^1^ Faculty of Medicine and Health Sciences University of East Anglia Norwich UK; ^2^ Department of Psychology Nottingham Trent University Nottingham UK; ^3^ Surrey Sleep Research Centre University of Surrey Guildford UK; ^4^ UK Dementia Research Institute University of Surrey Guildford UK

**Keywords:** circadian, diurnal preference, insomnia, sex differences

## Abstract

Sleep and circadian rhythms are considered to be important determinants of mental and physical health. Epidemiological studies have established the contribution of self‐reported sleep duration, sleep quality and chronotype to health outcomes. Mental health and sleep problems are more common in women and men are more likely to be evening types. Few studies have compared the relative strength of these contributions and few studies have assessed these contributions separately in men and women. Furthermore, sleep and circadian characteristics are typically assessed with a limited number of instruments and a narrow range of variables is considered, leaving the understanding of the relative contribution of different predictors somewhat fractionary. We compared sleep quality, sleep duration and chronotype as predictors for self‐reported mental and physical health and psychological characteristics in 410 men and 261 women aged 18 to 30. To ascertain that results were not dependent on the use of specific instruments we used a multitude of validated instruments including the Morningness‐Eveningness‐Questionnaire, Munich‐ChronoType‐Questionnaire, Pittsburgh‐Sleep‐Quality‐Index, British‐Sleep‐Survey, Karolinska‐Sleep‐Diary, Insomnia‐Severity‐Index, SF‐36‐Health Survey, General‐Health‐Questionnaire, Dutch‐Eating‐Behaviour‐Questionnaire, Big‐Five‐Inventory, Behaviour‐Inhibition‐System‐Behaviour‐Activation‐System, and the Positive‐Affect‐Negative‐Affect‐Schedule. Relative contributions of predictors were quantified as local effect sizes derived from multiple regression models. Across all questionnaires, sleep quality was the strongest independent predictor of health and in particular mental health and more so in women than in men. The effect of sleep duration and social jetlag was inconspicuous. A greater insight into the independent contributions of sleep quality and chronotype may aid the understanding of sleep‐health interactions in women and men.

## 1 INTRODUCTION

Sleep problems are common in people with mental health disorders (Freeman et al., 2017). While disrupted sleep was previously thought to be a consequence of mental health disorders, sleep problems are now being increasingly recognised as an important element of the complex and multi‐factorial causation of the symptoms and functional disability associated with psychiatric disorders (Harvey, Murray, Chandler, & Soehner, 2011).

Common sleep complaints relate to sleep initiation, maintenance, timing, duration and quality. Many surveys focusing on physical health outcomes have emphasized the importance of self‐reported sleep duration although the importance of self‐reported sleep quality has not gone unnoticed (Cappuccio, D'Elia, Strazzullo, & Miller, 2010; Dijk, 2012; Gallicchio & Kalesan, 2009; Magee, Kritharides, Attia, McElduff, & Banks, 2012).

Sleep duration has been assessed by a single question about habitual sleep duration (e.g. Pittsburgh Sleep Quality Index [PSQI]) (Buysse, Reynolds, Monk, Berman, & Kupfer, 1989), British Sleep Survey [BSS] (Groeger, Zijlstra, & Dijk, 2004)) and by separately assessing bedtime and wake time (PSQI, BSS and the Munich Chronotype Questionnaire [MCTQ] (Roenneberg, Wirz‐Justice, & Merrow, 2003)). Some of the questionnaires distinguish between workdays and free‐days (e.g. MCTQ, BSS). The Karolinska Sleep Diary (KSD) measures sleep‐wake timing for the last night only but it is often used over a longer time period (Akerstedt, Hume, Minors, & Waterhouse, 1994).

Sleep quality is often assessed by the total score of the PSQI, which is one of the most widely used questionnaires. The PSQI consists of 24 items and 7 domains and the single sleep quality question of the PSQI has also been used to assess subjective sleep quality. The KSD measures sleep quality of the previous night by one question. Another measure of sleep quality is the Insomnia Severity Index (ISI) primarily used in insomnia research but now also used outside insomnia research (Bastien, Vallieres, & Morin, 2001; Lazar et al., 2013).

Sleep duration and quality have both been associated with a variety of physical and mental health outcome measures (Baum et al., 2014; Cappuccio et al., 2010; Freeman et al., 2017; Gallicchio & Kalesan, 2009; Roberts & Duong, 2017), wellbeing (Wakefield, Bowe, Kellezi, Butcher, & Groeger, 2019), affect/ mood (Ong, Carde, Gross, & Manber, 2011), diet (Chaput, 2014; St‐Onge, Mikic, & Pietrolungo, 2016), and personality (Duggan, Friedman, McDevitt, & Mednick, 2014).

In recent years there has been increasing interest in the contribution of circadian disturbances and individual differences in circadian rhythmicity to mental and physical health. Circadian processes may contribute to health through their role in sleep regulation or through the separate wide range of biological and behavioural processes that are influenced by circadian rhythmicity (Logan & McClung, 2019).

Individual differences in circadian rhythms are often quantified by questionnaires assessing the preferred or actual timing of daily activities (e.g. sleep, physical activity). Based on these ‘chronotypes’ or ‘diurnal preference’ groups such as morning, intermediate and evening types can be identified. Chronotype has traditionally been measured with the Morningness‐Eveningness Questionnaire (MEQ) (Horne & Ostberg, 1976) and more recently with the MCTQ. A large volume of research indicates that evening‐type preference may be associated with poorer health outcomes, including mortality (Knutson & von Schantz, 2018; Paudel et al., 2010), diabetes (Yu et al., 2015), BMI (Baron, Reid, Kern, & Zee, 2011), obesity (Antunes Lda, Jornada, Ramalho, & Hidalgo, 2010), depression (Merikanto et al., 2015; Van den Berg, Kivela, & Antypa, 2018erg, Kivela, & Antypa, 2018), anxiety disorders (Park et al., 2015) and bipolar disorder (Gershon et al., 2018).

The effect of chronotype on mental health and well‐being has been suggested to be due to the impact of an evening‐type preference on sleep quality and sleep duration. Evening‐type adults commonly complain of decreased subjective sleep quality, insufficient sleep, excessive day time sleepiness and trouble initiating sleep (Kivela, Papadopoulos, & Antypa, 2018). Evening chronotype, low sleep quality and excessive daytime sleepiness have been shown to independently predict common mental disorders (Rose et al., 2015). Chronotype has also been related to well‐being (Drezno, Stolarski, & Matthews, 2019), personality, particularly Extraversion and sensation seeking (Caci, Robert, & Boyer, 2004; Kerkhof, 1985; Lipnevich et al., 2017), diet (Maukonen et al., 2016; Munoz, Canavate, Hernandez, Cara‐Salmeron, & Morante, 2017), and affect or mood (Jeong Jeong et al., 2015).

Eveningness has been associated with increased social jet leg (Roenneberg, Pilz, Zerbini, & Winnebeck, 2019). Social jetlag refers to a misalignment between biological time and sleep timing imposed by social schedules such as school and work times. Social jetlag may contribute to emerging mental health difficulties especially in adolescents and young adults (Doi, Ishihara, & Uchiyama, 2015). However, the association of social jetlag with mental health in young people is equivocal according to a recent systematic review (Henderson, Brady, & Robertson, 2019).

Despite the apparently overlapping effects of sleep and chronotype the independent contribution of sleep duration, sleep quality, chronotype and social jet lag on physical and mental health has rarely been assessed in surveys in which all three aspects were covered simultaneously and with multiple validated instruments. Furthermore in most studies reporting on associations between sleep and health outcomes the results are controlled for sex effects. Few studies have investigated sex specific associations between chronotype, sleep duration and sleep quality and mental health. Women have a greater risk of developing mental health conditions while a greater proportion of evening‐types are men (Antypa, Vogelzangs, Meesters, Schoevers, & Penninx, 2016). Although there are other factors associated with mental health to consider, from a sleep perspective, this presents a discrepancy in the link between mental health and the evening chronotype. Sex differences in the association between sleep and health deserve further exploration as women sleep longer than men and epidemiological studies suggest that poor sleep and sleep‐related problems are more strongly associated with poor health outcomes in women than in men (Lauderdale et al., 2006; Suarez, 2008). Women who report ‘unhealthy’ sleep have greater psychological distress and risk of cardiovascular disease, type 2 diabetes, depression and mood disorders (Suarez, 2008). Additionally, daytime sleepiness and poor sleep quality is higher among women, and female predominance in the rate of depression was observed in subjects with delayed sleep‐wake schedule (Fabbian et al., 2016).

These combined issues are addressed in the study reported below.

### 1.1 Aims and Significance

The major aim of this study was to establish whether chronotype, sleep quality and sleep duration, assessed with a number of frequently used instruments, are independent predictors of physical and mental health (e.g. affect, wellbeing), lifestyle (e.g. diet), and if so, which is the strongest predictor. The second aim was to investigate the independent associations of chronotype, sleep quality and sleep duration with stable trait‐like psychological characteristics. A third aim of this study was to assess how sex modulates the associations between chronotype, sleep quality, sleep duration and physical and mental health and psychological characteristics.

## 2 METHODS

### 2.1 Participants

The study was reviewed and approved by the University of Surrey Ethics Committee and conducted in accordance with the principles of the Declaration of Helsinki. Participants were recruited using posters, advertisements in local newspapers, by radio, and through Web sites. Following a 10 min long telephone interview to check for basic eligibility criteria 677 participants attended the face‐to‐face screening session at Surrey Clinical Research Centre (SCRC) and 675 participants completed the session, which lasted for approximately two hours and included 15 questionnaires and two paper based tests. Approximately 99% of the participants completed all questionnaires. In total 671 participants were included in the current analyses. For some questionnaires with derived outcome measures (e.g. global sleep quality measured by PSQI) the number of observations included in the analyses was lower because some questions were not answered. Participants were healthy, non‐smokers, aged between 20 and 35 years. We only studied young adults to minimise potential confounding effects of age. The participants did not do any shift‐work during year preceding the study and had a BMI between 18 and 30. There was no exclusion criteria related to ethnicity or country of origin.

The data were collected as part of the screening for a sleep‐circadian experiment and participants were reimbursed for reasonable out of pocket expenses, e.g. bus, train fares, etc and paid 10 pounds for their time. For additional details, please see the supplementary material and our previous report on this data set (Lazar et al., 2012).

### 2.2 Health and sleep measures

Chronotype, habitual sleep‐wake timing and quality of sleep, as well as psychological and self‐reported health characteristics were assessed by multiple questionnaires and scales (see Supplementary material for more details).

*Demographic data*, such as age, sex, BMI, income, ethnicity, marital status, etc., were assessed by a medical questionnaire (MQ) and the BSS.

*Diurnal preference (chronotype)* measures were taken from the total score over all the 19 items of the MEQ (Horne & Ostberg, 1976), the midpoint of sleep during free days (MSF) and the self‐assessment questions referring to being early or late type using the 7‐point Likert scale (MCTQ_Iam_) from the MCTQ questionnaire.

*Sleep‐wake timing and duration* were assessed using multiple questionnaires that differed from each other with respect to the time period to which they refer (MQ – in general, PSQI – last month, BSS – last week, and MCTQ – usual workdays and free days). PSQI and BSS were used to provide a direct measure of self‐reported sleep duration and the MCTQ was used for the assessment of both sleep‐wake timing and sleep duration. Here we extracted measures of self‐reported bedtime (BT) and wakeup time (WT), and sleep latency (SL). From these self‐reported measures, we derived some further sleep timing parameters: time in bed (TIB), sleep onset time (SOT), sleep period time (SPT) and midpoint of sleep (MS). TIB, the total time spent in bed, was calculated as the total hours between bedtime and wakeup time. SOT, the time of actual sleep onset, was calculated by adding the sleep latency time (time it takes to fall asleep) to bedtime. SPT, the total time spent sleeping, was calculated as the total hours between sleep onset time and wakeup time and was used as a measure of sleep duration. MS was calculated as the clock time corresponding to the half of SPT. As the MCTQ provided separate work‐ and free‐day data, this allowed for separate and average calculations of the measures discussed above. This average was weighted by number of working days and free days reported by the participants (Allebrandt et al., 2010), e.g., BTA = (BTW × WDn +BTR × RDn)/7, where BTA = average bedtime, BTW = bedtime on workdays, WDn = number of workdays, BTR = bedtime on free days, and FDn = number of free days. MS during free days (MSF) when used as a chronotype indicator was also corrected for the sleep debt accumulated during the week according to the following formula: MSFcorr = MSF−0.5×(TIBF−[TIBW × WDn+TIBF × FDn]/7), where MSFcorr = corrected MS during free days, TIBW = TIB on workdays, WDn = number of workdays, TIBF = TIB on free days, and FDn = number of free days (Roenneberg et al., 2004).

*Quality of sleep* was assessed by the ISI total score across all the seven items (Bastien et al., 2001), the KSD single sleep quality question measured on 9‐point Likert scale (‘How would you rate your quality of sleep?’ ‐ referring to the last night (Akerstedt et al., 1994), the PSQI global score across all the 24 items and the single sleep quality question within the PSQI measured on a 4‐point Likert scale (‘During the past month, how would you rate your sleep quality overall?’) (Buysse et al., 1989).

Social jetlag was computed as the absolute difference between the uncorrected midpoint of sleep during free‐days and workdays.

For further details on the sleep‐timing outcome measures please refer to the supplementary material.

*Health profile* was assessed using Version 2 of the 36‐Item Short‐Form Health Survey (SF‐36v2) which provides a separate measure for physical and mental health (Ware & Sherbourne, 1992). The SF‐36 has been previously used to investigate sleep and health associations in healthy young adults (Gulec et al., 2013). The General Health Questionnaire (GHQ) provides a composite total score as a reliable measure for general psychiatric health (Goldberg, 1978). In this way mental health was directly measured by two different questionnaires the SF36 and the GHQ, but labelled as mental health and general psychiatric health, respectively. BMI was also included as a proxy measure of physical health.

*Eating behaviour* was assessed using the English version of the Dutch Eating Behaviour Questionnaire (DEBQ) (Van Strien, Frijters, Bergers, & Defares, 1986).

*Personality* was measured using the Big Five Inventory (BFI) (Goldberg, 1990).

*Individual traits of general motivation systems* were assessed by the Behavioural Inhibition System–Behavioural Activation System (BIS‐BAS) scales (Carver & White, 1994).

*General affective orientation* reported for the last couple of weeks was measured by the Positive Affect and Negative Affect Schedule (PANAS) (Watson, Clark, & Tellegen, 1988).

### 2.3 Statistical analysis

Statistical analyses were conducted using SPSS (version 25) and SAS (version 9.4).

In a first step for assessment of sex differences amongst demographic, sleep and health measures simple *t*‐tests were used (Table 1). For measures that were grouped categorical data, a chi‐square test was used.

**Table 1:** Sex Differences Across the Studied Demographic, Sleep and Health Measures

Studied Variables	Men			Women			Main effect			q‐value
	*N*	Mean	SD	*N*	Mean	SD	t	df	P‐value	
Age (yrs) *r=(18‐36)*	410	25.23	4.18	261	25.85	4.10	−1.89	669.00	0.059	0.127
*Ethnic Group:*										
European	287	‐	‐	181	‐	‐	*Chi Squared:*	5	0.084	0.164
African	49	–	–	41	–	–	9.71^a^			
Indian	34	–	–	9	–	–				
Oriental	19	–	–	12	‐	‐				
Other	9	–	–	11	‐	‐				
No answer	12	–	–	7	‐	‐				
*Gross Income:*										
Prefer not to say	154	‐	‐	112	‐	‐	*Chi Squared:*	1	0.192	0.296
Data provided	247	‐	‐	144	‐	‐	1.853^a^		0.374	0.470
		‐	‐		‐	‐				
*Marital Status*:										
Lives with partner	101	‐	‐	70	‐	‐	*Chi Squared:*	1	0.466	0.547
Lives alone	306	‐	‐	185	‐	‐	0.568^a^			
*Work Status:*							*Chi Squared:* 1.563^a^	1	0.231	0.345
Working/in education	320	‐	‐	216	‐	‐				
Currently not working	86	‐	‐	45	‐	‐				
Alcohol Intake (units per week) *r = (0‐24)*	395	4.86	4.42	260	3.46	3.30	4.63	642.36	**<0.0001**	**0.004**
*Chronotype*										
MSFcorr (MCTQ)^a^ *r=(0:33‐7:58)*	410	04:41	1:10	261	04:31	1:02	1.82	597.12	0.069	0.188
MEQ score *r=(25‐73)*	401	50.53	8.25	258	50.97	7.64	−0.69	657.00	0.490	0.567
1‐morning	66	‐	‐	38	‐	‐	*Chi Squared:*	2	0.027	0.070
2‐intermediate	271	‐	‐	195	‐	‐				
3‐evening	73	‐	‐	28	‐	‐	7.25^a^			
MCTQ_Iam_ *r=(1‐6)*	405	3.06	1.53	261	3.07	1.59	−0.01	664.00	0.994	0.994
*Health measures*										
Psychiatric Health (GHQ) *r=(0‐34)*	405	8.05	3.33	257	8.54	3.75	−1.76	660.00	0.079	0.162
Mental Health (SF36) *r=(44‐100)*	410	85.54	8.55	261	83.10	10.53	3.15	471.28	**0.002**	**0.007**
Physical Health (SF36) *r=(48‐100)*	410	92.89	7.17	261	92.30	8.35	0.98	669.00	0.330	0.433
BMI (kg/m^2^) *r=(17‐38)*	404	23.88	2.67	256	22.71	3.03	5.08	491.54	**<0.0001**	**0.002**
*Sleep quality*										
ISI *r=(0‐21)*	399	3.16	2.99	249	3.57	3.39	−1.61	646.00	0.107	0.185
PSQI_g_ *r=(0‐11)*	372	3.22	1.83	246	3.07	1.81	0.95	616.00	0.344	0.445
PSQI_sq_ *r=(0‐3)*	409	0.58	0.54	260	0.55	0.56	0.14	667.00	0.408	0.506
KSD *r=(1‐8)*	406	3.56	1.46	255	3.33	1.30	2.17	584.82	0.031	0.078
*MCTQ workdays (general)*										
Bedtime^a^ *r=(22:00‐3:00)*	406	23:25	1:06	260	23:04	0:53	4.27	599.84	**<0.0001**	**0.001**
Sleep latency^b^ *r=(0‐120)*	405	16.8	11.4	261	16.2	12.0	0.45	664.00	0.656	0.722
Sleep onset time^a^ *r=(22:00‐3:00)*	405	23:42	1:02	260	23:22	0:54	4.29	602.04	**<0.0001**	**0.001**
Midpoint of sleep^a^ *r=(00:15‐7:31)*	401	03:46	1:01	260	03:32	0:52	3.29	609.41	**0.001**	**0.004**
Wakeup time^a^ *r=(3:00‐12:00)*	401	07:50	1:13	260	07:41	1:07	1.75	659.00	0.080	0.160
Time in bed^c^ *r=(5:00‐12:00)*	401	8:26	1:03	260	8:35	1:05	−1.72	659.00	0.088	0.168
*MCTQ free days (general)*										
Bedtime^a^ *r=(21:00‐4:00)*	410	00:19	1:10	261	00:05	1:07	2.60	577.31	**0.010**	**0.029**
Sleep latency^b^ *r=(0‐60)*	410	15.0	11.4	261	13.6	10.2	1.02	669.00	0.307	0.416
Sleep onset time^a^ *r=(21:00‐4:00)*	410	00:34	1:10	261	00:19	1:07	2.74	669.00	**0.006**	**0.018**
Midpoint of sleep^a^ *r=(1:15‐9:02)*	410	04:44	1:11	261	04:36	1:01	1.62	610.58	0.107	0.181
Wakeup time^a^ *r=(5:00‐14:00)*	410	08:54	1:28	261	08:52	1:19	0.26	669.00	0.799	0.837
Time in bed^c^ *r=(3:00‐12:00)*	410	8:35	1:14	261	8:47	1:22	−2.00	669.00	0.046	0.107
MCTQ Average (general)										
Sleep duration^c^ *r=(4:33‐11:50)*	392	8:14	1:01	257	8:25	1:01	−2.12	647.00	0.034	0.081
*MCTQ change*										
Bedtime^c^ *r=(‐3:00‐4:00)*	406	0:54	0.53	260	0:59	0:52	−1.26	664.00	0.207	0.314
Sleep latency^b^ *r=(‐90‐30)*	405	−1.8	7.2	261	−2.4	9.0	0.77	664.00	0.443	0.527
Sleep onset time^c^ *r=(‐3:05‐4:00)*	405	0:52	0:52	260	0:57	0:52	−1.13	663.00	0.259	0.380
Midpoint of sleep^c^ *r=(‐1:30‐4:33)*	401	0:58	0:53	260	1:04	0:50	−1.52	659.00	0.128	0.209
Wakeup time^c^ *r=(‐3:00‐6:00)*	401	1:03	1:14	260	1:11	1:16	−1.41	659.00	0.159	0.250
Time in bed^c^ *r=(‐4:00‐5:00)*	401	0:08	1:11	260	0:12	1:22	−0.59	496.42	0.556	0.627
*DEBQ*										
Restrained Eating *r=(1.0‐4.6)*	403	1.99	0.74	259	2.53	0.78	−9.06	660.00	**<0.0001**	**0.001**
Emotional Eating *r=(1.0‐4.6)*	405	1.77	0.62	259	2.21	0.80	−7.50	455.04	**<0.0001**	**0.001**
External Eating *r=(1.2‐5.0)*	407	3.03	0.65	259	3.05	0.66	−0.36	664.00	0.717	0.779
*BFI*										
Openness *r=(21‐50)*	402	38.49	5.01	255	38.36	5.43	0.32	508.21	0.752	0.797
Conscientiousness *r=(19‐45)*	403	33.66	5.43	250	34.99	5.58	−3.01	651.00	**0.003**	**0.009**
Extraversion *r=(14‐40)*	405	28.64	4.81	257	29.40	5.32	−1.90	660.00	0.058	0.128
Agreeableness *r=(19‐45)*	405	35.35	4.86	255	37.05	4.50	−4.57	570.57	**<0.0001**	**0.001**
Neuroticism *r=(8‐35)*	404	17.12	5.29	257	19.44	5.36	−5.48	659.00	**<0.0001**	**0.001**
*PANAS*										
Positive Affect *r=(14‐50)*	405	37.74	6.01	255	37.23	6.33	1.04	658.00	0.297	0.422
Negative Affect *r=(10‐38)*	402	15.48	5.05	253	16.09	4.96	−1.51	653.00	0.131	0.210
*BIS‐BAS*										
BIS *r=(7‐28)*	400	17.46	3.44	256	15.00	3.29	9.10	654.00	**<0.0001**	**0.000**
BAS reward *r=(5‐15)*	401	7.89	1.92	255	7.40	1.87	3.17	654.00	**0.002**	**0.007**
BAS drive *r=(4‐16)*	401	8.36	2.17	258	8.35	2.12	0.05	657.00	0.964	0.975
BAS fun seeking *r=(4‐13)*	403	6.92	1.97	258	7.07	2.13	−0.93	659.00	0.354	0.451

Abbreviations: MSFcorr = Midpoint of sleep during free days corrected for the sleep debt accumulated during the week, MEQ = Morningness‐Evenigness Questionnaire, MCTQ = Munich Chronotype Questionnaire, DEBQ = Dutch Eating Behaviour Questionnaire, BFI= Big Five Inventory, PANAS = Positive and Negative Affect Schedule, BIS‐BAS= Behavioural Inhibition and Approach Systems questionnaire, PSQI = Pittsburgh Sleep Quality Index, KSD = Karolinska Sleep Diary, ISI = Insomnia Severity Index, r = range . For further details on the reported outcome meaures please see methods.

Sex differences are FDR corrected. Only bold P values reach significance after statistical correction.

Time units = ^a^clock time, ^b^minutes, ^c^hours:minutes; q value = FDR adjusted significance level (see methods).

In a second step regression models were applied for the assessment of the independent associations of chronotype, sleep quality and sleep duration on health and psychological wellbeing measures. Analyses were performed with and without control for sex, age, ethnicity, work status, gross income, marital status and alcohol consumption (Table 2). Multicollinearity was assessed by examining tolerance and the Variance Inflation Factor (VIF) (Table S1). For more details on the multivariate regression models please refer to the supplementary material.

**Table 2:** The Independent Contribution of Chronotype, Sleep Quality and Sleep Duration on Measures of Health and Psychological Characteristics

Physical and Psychological Wellbeing Variables	Chronotype				Sleep Quality				Sleep Duration				
	Beta (95% CI)	p‐value	p‐value controlled	*f^2^*	Beta (95% CI)	p‐ value	p‐value controlled	*f^2^*	Beta (95% CI)	p‐value	p‐value controlled	*f^2^*	Adjusted R Square Without (with covariates)
Psychiatric Health	−0.079 (−0.114, −0.044)	**<0.0001**	**<0.0001**	0.033	0.457 (0.303, 0.611)	**<0.0001** ^abc^	**<0.0001**	0.058	0.116 (−0.153, 0.384)	0.398	0.389	0.001	0.099 (0.109)
Mental Health	0.170 (0.080, 0.259)	**<0.0001**	**<0.0001**	0.024	−1.882 (−2.274, −1.491)	**<0.0001** ^abc^	**<0.0001**	0.152	−0.071 (−0.753, 0.611)	0.837	0.825	0.000	0.168 (0.179)
Physical Health	0.002 (−0.072, 0.076)	0.951	0.984	0.000	−1.283 (−1.607, −0.959)	**<0.0001** ^abc^	**<0.0001**	0.103	−0.080 (−0.644, 0.485)	0.782	0.915	0.000	0.093 (0.106)
BMI	0.006 (−0.024, 0.036)	0.690	0.910	0.000	0.076 (−0.055, 0.207)	0.253	0.274	0.002	−0.159 (−0.388, 0.070)	0.173	0.679	0.003	0.001 (0.048)
*DEBQ*													
Restrained Eating	0.007 (−0.001, 0.016)	0.084	0.198	0.005	0.029 (−0.008, 0.065)	0.121^a^	**0.048**	0.004	0.024 (−0.039, 0.088)	0.450	0.897	0.001	0.003 (0.122)
Emotional Eating	−0.010 (−0.018, −0.003)	**0.007**	**0.004**	0.013	0.026 (−0.007, 0.058)	0.122^a^	0.062	0.004	−0.017 (−0.074, 0.041)	0.570	0.235	0.001	0.015 (0.123)
External Eating	−0.016 (−0.023, −0.009)	**<0.0001**	**<0.0001**	0.038	0.040 (0.011, 0.069)	**0.007** ^a^	**0.014**	0.013	−0.016 (−0.067, 0.034)	0.530	0.386	0.001	0.054 (0.066)
*BFI*													
Openness	−0.045 (−0.099,0.010)	0.107	0.135	0.005	−0.123 (−0.360, 0.115)	0.311^a^	0.315	0.002	−0.253 (−0.669, 0.163)	0.232	0.296	0.002	0.002 (0.009)
Conscientiousness	0.231 (0.178, 0.285)	**<0.0001**	**<0.0001**	0.127	−0.294 (−0.532,−0.057)	**0.015** ^ac^	**0.029**	0.010	0.224 (−0.190, 0.638)	0.289	0.348	0.002	0.132 (0.162)
Extraversion	0.054 (0.002,0.105)	**0.040**	**0.011**	0.007	−0.284 (−0.509,−0.059)	**0.013** ^ab^	**0.005**	0.011	0.064 (−0.330, 0.459)	0.748	0.982	0.000	0.017 (0.039)
Agreeableness	0.096 (0.048,0.143)	**<0.0001**	**0.002**	0.027	−0.434 (−0.644, −0.223)	**<0.0001** ^abc^	**0.000**	0.028	0.418 (0.052, 0.784)	0.025	0.098	0.009	0.066 (0.109)
Neuroticism	−0.105 (−0.159,−0.051)	**<0.0001**	**<0.0001**	0.025	0.696 (0.461, 0.930)	**<0.0001** ^abc^	**<0.0001**	0.058	0.120 (−0.292, 0.531)	0.568	0.498	0.001	0.090 (0.158)
*PANAS*													
Positive Affect	0.155 (0.094, 0.217)	**<0.0001**	**<0.0001**	0.043	−0.422 (−0.691, −0.152)	**0.002** ^abc^	**0.003**	0.016	−0.366 (−0.838, 0.105)	0.128	0.181	0.004	0.068 (0.062)
Negative Affect	−0.055 (−0.106,−0.004)	**0.036**	**0.018**	0.008	0.544 (0.320, 0.767)	**<0.0001** ^abc^	**<0.0001**	0.040	−0.020 (−0.415, 0.374)	0.920	0.966	0.000	0.049 (0.089)
*BIS‐BAS*													
BIS	0.034 (−0.003, 0.071)	0.072	**0.011**	0.006	−0.235 (−0.397, −0.073)	**0.005** ^ab^	**0.001**	0.014	−0.054 (−0.339, 0.231)	0.711	0.835	0.000	0.019 (0.147)
BAS reward	−0.018 (−0.038,0.003)	0.086	0.124	0.005	−0.016 (−0.103, 0.072)	0.727^c^	0.880	0.000	0.022 (−0.132, 0.176)	0.779	0.676	0.000	0.000 (0.028)
BAS drive	−0.030 (−0.052,−0.007)	**0.010**	**0.022**	0.012	0.088 (−0.010, 0.186)	0.078^c^	0.059	0.005	0.032 (−0.139, 0.202)	0.717	0.985	0.000	0.016 (0.022)
BAS fun seeking	0.022 (0.000,0.043)	**0.045**	0.667	0.007	0.013 (−0.080, 0.106)	0.789^b^	0.328	0.000	0.015 (−0.148, 0.179)	0.855	0.684	0.000	0.002 (0.086)

Note

Regression model including chronotype, sleep quality and sleep duration as predictors and measures of physical and mental health and psychological wellbeing as dependent variables. Chronotype is measured as the total score of the Morningness‐Evenigness Questionnaire (MEQ) where higher score indicates greater morning preference. Sleep quality is measured as the global score from the Pittsburgh Sleep Quality Index (PSQI), where a higher score indicates poorer sleep quality. Sleep duration is measured as the average sleep period time across work days and free days from the Munich Chronotype Questionnaire (MCTQ) where an increasing score indicates a longer sleep duration (see methods). Higher scores of general psychiatric health (measured by the General Health Questionnaire) indicate poorer health, whilst lower scores in physical and mental health as measured by the 36‐Item Short‐Form Health Survey (SF‐36) indicate poorer health outcomes. For the Dutch Eating Behaviour Questionnaire (DEBQ) higher scores indicate greater endorsement of the eating behaviour, for the Big Five Inventory (BFI) a higher score indicates the stronger presence of a personality trait, the Positive and Negative Affect Schedule (PANAS) higher scores indicate greater positive or negative engagement with the environment, and for the Behavioural Inhibition and Approach systems questionnaire (BIS‐BAS) lower scores indicate a greater drive through the anticipation of either punishment or reward. Second *p* value indicates significance after controlling for additional covariates: *sex, age, ethnicity, work status, gross income, marital status and alcohol consumption*. P values in bold indicate significant effects (*p* < .05). Superscript letters represent whether statistically corrected p‐values were significant when using other measures than PSQI global score as sleep quality indicators: ^a^Insomnia Severity Index (ISI) total score; ^b^PSQI single sleep quality question (a 4‐point Likert scale ‐ ‘During the past month, how would you rate your sleep quality overall?’); ^c^Karolinska Sleep diary (KSD) single sleep quality question (a 9‐point Likert scale ‐ ‘How would you rate your quality of sleep?’ – referring to the last night). According to Cohen's (1988) guidelines f^2^ ≥ 0.02, f^2^ ≥ 0.15, and f^2^ ≥ 0.35 represent small, medium, and large effect sizes, respectively.

The independent effect of chronotype, sleep duration and sleep quality on physical and mental health and psychological wellbeing was estimated by Local effect size calculations of Cohen's *f* ^2^. This quantifies the proportion of variance explained by adding a sleep or chronotype predictor to the model with confounders alone (Selya, Rose, Dierker, Hedeker, & Mermelstein, 2012).

In order to further explore sex differences in the association between chronotype, sleep quality, sleep duration and the main three health outcome measure we computed Pearson correlations separately for men and women and compared the correlation coefficients using Fisher's transformation. Additionally, the local effect size for the associations of chronotype, sleep duration and the four different sleep quality measures with general psychiatric, mental and physical health for each sex group was calculated. Finally, we also assessed the independent contribution of social jetlag to measures of health and psychological well‐being.

In order to address multiplicity we estimated false discovery rate analysis using the Benjamini‐Hochberg correction (Benjamini & Hochberg, 1995). The False discovery rate was set at 0.05 and significance was established if the q values were smaller than 0.05. For the regression analyses we did not apply FDR correction. This is because the p values associated with the individual predictors cannot be considered independent within the same multivariate model.

## 3 RESULTS

### 3.1 Sex differences across demographic, sleep and health measures

Demographic data did not show any significant differences between men and women, except for alcohol intake which indicated that men drink significantly more (~1.4 unit more) per week than women (Table 1).

#### 3.1.1 Sleep measures

Women had significantly earlier bedtime and sleep onset time during both workdays and free‐days, as measured by the MCTQ. Women also reported a significantly earlier midpoint of sleep during workdays (MSW) than men, but this effect was present only as a trend during free‐days. Women spent more time in bed throughout the week and mainly during free‐days, and they also reported an overall worse sleep quality as measured by KSD single sleep quality question compared to men. Nonetheless, these sex effects on time in bed during free‐days and sleep quality lost significance after statistical correction for multiplicity (Table 1).

#### 3.1.2 Health/Psychological/Personality measures

Women had significantly poorer mental health and lower BMI scores. Women were significantly more likely to eat in response to emotional challenges and required significantly greater effort to refrain from eating. Women were significantly more Conscientious, Agreeable and inclined towards Neuroticism. Men were significantly more driven in anticipation of rewards and to avoid punishment.

### 3.2 The effect of chronotype, sleep quality and sleep duration associations on health and psychological measures

In the second step, we analysed the associations between chronotype, sleep quality and sleep duration with health and psychological measures. This was repeated with covariates of sex, age, ethnicity, work status, gross income, marital status and alcohol consumption included in the model (Table 2).

Chronotype and sleep quality were found to predict independently multiple health and psychological wellbeing measures across all participants whereas sleep duration did not yield a significant effect on any of the outcome measures. Poorer general psychiatric health and mental health were independently predicted by a later chronotype and worse sleep quality. In contrast, reduced physical health was only significantly predicted by poor sleep quality.

Similarly, later chronotype and poor sleep quality independently predicted numerous other psychological proxies for mental health. This included unhealthy eating behaviour (increased external and emotional eating), a weaker presence of favourable (Conscientiousness, Extraversion, Agreeableness) and stronger presence of unfavourable personality traits for mental health (neuroticism), decreased positive and increased negative engagement with the environment (positive effect and negative effect) as well as a greater drive through anticipation of punishment (BIS). Most effects were relatively strong and prevailed after control for further confounding factors (sex, age, ethnicity, work status, gross income, marital status and alcohol) (Table 2). Interestingly, sleep duration did not predict any of studied health and psychological outcome measures except agreeableness which was independently associated with longer sleep duration.

**Figure 1:**
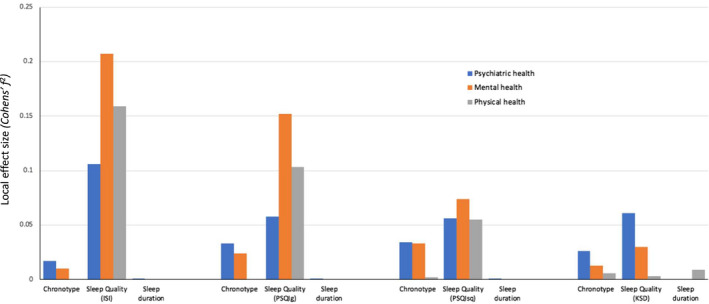
The Effect of Chronotype, Sleep Duration and four different Sleep Quality measures on Health. A visual summary of the local effect sizes of chronotype, sleep duration and different sleep quality measures on the three main health outcomes. The chronotype measure is based on total scores of the Morningness‐Evenigness Questionnaire (MEQ), the sleep duration measure is based on the average sleep period time calculation from the Munich Chronotype Questionnaire (MCTQ) and the sleep quality measures are from the Insomnia Severity Index (ISI), global score and single sleep quality question from the Pittsburgh Sleep Quality Index (PSQI) and the single sleep quality question from the Karolinska Sleep Diary (KSD). According to Cohen's (1988) guidelines *f^2^* ≥ 0.02, *f^2^* ≥ 0.15, and *f^2^* ≥ 0.35 represent small, medium, and large effect sizes, respectively

The relative strength of the associations of chronotype, sleep quality and sleep duration predictor variables with the three main health related dependent variables was quantified by local effect sizes. Figure 1 shows the local effect size of the three predictor variables, chronotype as measured by the MEQ, sleep duration as measured by the MCTQ and sleep quality as measured by three different questionnaires (ISI, PSQI and KSD), on general psychiatric, mental and physical health dependent variables. The PSQI global score and the single PSQI sleep quality question were used as different sleep quality predictor variables in two separate models. Sleep quality emerges as the strongest predictor of health, with a particularly large effect on mental health. Among the four sleep quality measures ISI score represents the sleep quality proxy that best predicts all three health outcome measures (*f^2^* = 0.1091–0.2395) (Figure 1 and Table S2a). Chronotype is most strongly associated with general health whereas sleep duration associates most strongly with physical health (Figure 1 and Table 2).

To clarify the extent to which the results might be dependent on the individual questionnaires we ran the same analyses using all possible combinations of predictors across three chronotype (MEQ, MCTQ self‐assessment question [MCTQ_Iam_], and MCTQ midpoint of sleep during free‐days corrected for the sleep debt accumulated during the week [MSF_corr_]), three sleep duration (MQ, PSQI and MCTQ) and three sleep quality (ISI, PSQI global score and PSQI single sleep quality question) measures in 27 separate multivariate models.

**Figure 2:**
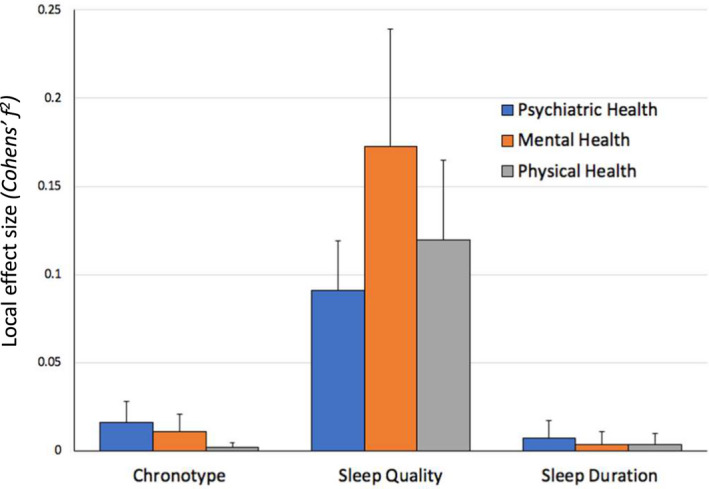
The Effect of Chronotype, Sleep Quality and Sleep Duration on Health Measures. A visual summary of the average local effect sizes of the chronotype, sleep quality, and sleep duration measures on general psychiatric, mental and physical health outcomes. Each column represents the average effect size of all chronotype, sleep quality and sleep duration measures from 27 different regression model combinations with the error bar representing one standard deviation. According to Cohen's (1988) guidelines *f^2^* ≥ 0.02, *f^2^* ≥ 0.15, and *f^2^* ≥ 0.35 represent small, medium, and large effect sizes, respectively

The results are consistent with the earlier findings; that is sleep quality has the strongest association with health factors (Tables S3a, b and c). The average local effect size of the associations of chronotype, sleep quality and sleep duration with general psychiatric, mental and physical health measures (Figure 2) are consistent with those presented in Table 2. Sleep quality presents the highest effect size (*f* ^2^ = 0.0907–0.1725) across the three health outcome measures followed by chronotype (*f* ^2^ = 0.0021–0.0161). Interestingly, sleep duration had no significant effect on any of the health outcome measures for any of the questionnaires (*f* ^2^ = 0.0034–0.0074). Chronotype was significantly associated with health outcomes when measured as diurnal preference (MEQ) and self‐assessed Morningenss‐Eveningness (MCTQ_Iam_) but not when estimated by the midpoint of sleep during free‐days corrected for the sleep debt accumulated during the week [MSF_corr_] as measured by the MCTQ (Tables S3a, b and c).

### 3.3 Sex differences in the effect of chronotype, sleep quality and sleep duration on health measures

**Figure 3:**
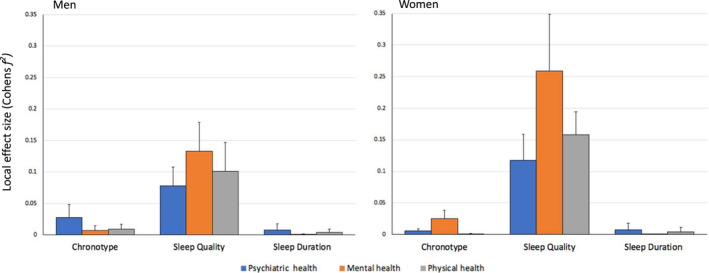
Sex Differences in the Effect of Chronotype, Sleep Quality and Sleep Duration on Health. A visual summary of the average local effect sizes of the chronotype, sleep quality, and sleep duration measures on general psychiatric, mental and physical health outcomes, with separate plots for men and women. Each column represents the average effect size of all chronotype, sleep quality and sleep duration measures from 27 different regression model combinations with the error bar representing one standard deviation. According to Cohen's (1988) guidelines *f^2^* ≥ 0.02, *f^2^* ≥ 0.15, and *f^2^* ≥ 0.35 represent small, medium, and large effect sizes, respectively

In the following step we aimed to clarify the extent to which the predictive power of chronotype, sleep quality, sleep duration on health may be different for men and women. We first ran a model in which we used all the four sleep quality measures as presented in Figure 1, but this time separately for men and women. Sleep quality was the strongest predictor of health across men and women, with significant effects for each sex group, however, effect size was considerably larger for women than for men, across all sleep quality measures (Figure S1 and Tables S4a–d). Mental health was also independently predicted by chronotype and significantly so for both men and women but with a stronger effect size in women. However, the effect of chronotype on general psychiatric health was significant in men only with a stronger effect as compared to women (Figure S1, Tables S4a–d). The average local effect size across the 27 multivariate models analysing the associations of chronotype, sleep quality and sleep duration with measures of mental and physical health also showed that the association between sleep quality and health factors is stronger in women (*f* ^2^ = 0.1178–0.2593) than in men (*f* ^2^ = 0.0782–0.1328), with the greatest difference seen in mental health as measured by the SF‐36 (Figure 3). Chronotype most strongly associated with general psychiatric health in men and mental health in women (Figure 3). The average effect size of sleep quality across the 27 models was significantly larger for women than for men for all three main health outcome measures (*p* < .05 after statistical correction). Sleep duration effects however, were largely non‐significant across men and women for the three main health outcome measures.

Comparing the correlations coefficients between chronotype, sleep quality, sleep duration and the three main health outcome measures showed that the relationship between sleep quality and mental health was significantly stronger in women than in men (Table S5).

### 3.4 The independent effect of social jet lag on measures of health and psychological characteristics

In a final step we conducted a non‐exhaustive analysis of the effect of social jetlag on measures of health and psychological characteristics independent of chronotype, sleep quality and duration. Social jetlag had a significant effect [β = −0.39(−0.71, −0.08), *p* = .013, *f^2^* = 0.010] on general psychiatric health measured by the General Health questionnaire (GHQ) with some marginal significant effects related to external eating and BAS fun seeking (Table S6). However, the independent effects of both Chronotype as measured in this model by the Morningness‐Eveningnes Questionnaire [β = −0.09(−0.12, −0.06), *p *< .0001, *f^2^* = 0.042] and Sleep quality as measured by the Self‐reported question from the PSQI [β = 1.50 (1.02, 1.99), *p *< .0001 *f^2^* = 0.059] had much greater significance level and larger effect size on psychiatric health. Although social jetlag overall showed slightly more effects then self‐reported sleep duration but remarkably less and weaker effects than self‐reported chronotype and sleep quality on outcome measures of health and psychological characteristics.

## 4 DISCUSSION

Our results based on numerous validated questionnaires and measures of chronotype, sleep quality and sleep duration show unequivocally that self‐reported sleep quality is the strongest predictor of mental and physical health and more so in women than in men.

Our results for sleep quality and mental health are in general in accordance with the depression and anxiety literature (Baglioni et al., 2011; Franzen & Buysse, 2008; Tafoya et al., 2019). However, in contrast to previous studies, our study did not show many effects of sleep duration on mental health (Table 2 and Tables S2a–c) and the local effect size of sleep duration was much smaller compared to the effect size of sleep quality and chronotype. Previous studies have reported associations between sleep duration and mental health (Baum et al., 2014; Roberts & Duong, 2017; Zhai, Zhang, & Zhang, 2015). It may be that in previous studies the association between sleep duration and mental health was mediated by effects on sleep quality and partly by chronotype.

This study found numerous significant associations between sleep quality and proxies of mental health. Positive affect and negative affect were shown to be associated with sleep quality in accordance with a previous study (Hoag, Tennen, Stevens, Coman, & Wu, 2016). Markarian, Pickett, Deveson, & Kanona, (2013) suggested that BIS sensitivity may be related to poor sleep quality, which is in support of the findings of the current study. Markarian and colleagues also reported that high BIS sensitivity is related to neuroticism which is the personality factor that had the strongest association with sleep quality in our study. Weaker but significant associations were found between Conscientiousness, Extraversion, Agreeableness and sleep quality. These associations between personality and sleep quality have been previously reported in the literature (Stephan, Sutin, Bayard, Krizan, & Terracciano, 2018). All of these personality factors have been shown to affect subjective well‐being, which is a significant topic in mental health research. Sleep quality has recently been identified as the mediator of this relationship (Lai, 2018).

Previous studies have demonstrated associations between both diurnal preference and chronotype with mental and physical health conditions (Knutson & von Schantz, 2018; Merikanto et al., 2015; Yu et al., 2015). Diurnal preference as measured by the MEQ correlates with chronotype as assessed by reported sleep timing. Diurnal preference correlates sleep‐wake timing, the phase of the melatonin rhythm and with circadian period (Hasan et al., 2012; Lazar et al., 2013). Our results indicate that self‐reported diurnal preference, but not chronotype, measured as mid‐point of sleep on free days, was associated with general and mental health. This may imply that participants were not sleeping and waking at preferred times. Not sleeping at preferred timings can impact sleep quality, particularly for evening‐type individuals (Gangwar et al., 2018) and this may explain the association between diurnal preference and not chronotype, with mental health. We did not find a major contribution of social jetlag to any of our outcome measures and this is in accordance with a recent systematic review (Henderson et al., 2019).

Morning preference was associated with stronger presence of favourable (Conscientiousness, Extraversion, Agreeableness) and weaker presence of unfavourable personality traits for mental health (neuroticism), which is in accordance with previous reports (Randler, Baumann, & Horzum, 2014).

In our study physical health was only associated with sleep quality. This is in disagreement with previous studies which found that physical health, which included increased risk of cardiovascular disease (Grandner, 2017; Merikanto et al., 2013), diabetes and metabolic syndrome (Yu et al., 2015), as well as higher caloric intake, BMI and rates of obesity (Arora & Taheri, 2015; Knutson, 2012) was also associated with chronotype and sleep duration. The reasons for this discrepancy may be because in our study the participants were young healthy adults in whom the impact of sleep duration and chronotype on physical health may not have yet manifested. Additionally, our study did not include smokers and those who reported to drink more than 14 alcohol units / week and numerous studies have shown that late chronotypes are more likely to smoke and have higher alcohol consumption (Wittmann, Paulus, & Roenneberg, 2010).

### 4.1 Identifying the strongest predictor of physical and mental health status

Effect size analysis identified sleep quality as the strongest predictor for all both mental health and physical health. This may appear to be in contrast to the literature which reports strong associations between chronotype and health factors. In fact, some studies suggest that chronotype can be used as a predictor for mental health disorders and should be considered in the development of mental health interventions (Taylor & Hasler, 2018). However, a recent study (Druiven et al., 2019) reports that a later chronotype does not predict a persistent course of depressive or anxiety disorders. Furthermore the association between chronotype and health factors could be mediated by the impact of a later chronotype on other sleep factors (Kivela et al., 2018). Simor, Zavecz, Palosi, Torok, & Koteles (2015) reported that insomnia complaints and disturbed sleep were mediators of negative psychological outcomes in evening‐type individuals and that chronotype only explained 6% of the variance of negative emotions while sleep problems accounted for a much larger proportion (28%). Moreover, another group has identified sleep quality as the only significant mediator between chronotype and depressive symptoms (Van den Berg et al., 2018).

### 4.2 Gender differences in the associations between sleep and health factors

Sleep quality associations were stronger in women while chronotype associations with general psychiatric health were only significant for men and chronotype associations with mental health were significant predominantly in women. There was also a significantly stronger correlation between chronotype and mental health in women compared to men. These results are in line with the literature. Eveningness was more significantly associated with impulsivity and anger, depression, anxiety disorders and low mood in women (Fabbian et al., 2016).

Our finding that sleep quality had a significantly stronger association with mental health in women than in men is also in accordance with the literature. Suarez (Suarez, 2008) reported that poor sleep quality and prolonged sleep latency incurred a greater psychological and physiological toll on women relative to men.

In our study, not all measures of sleep quality showed these relationships equally clearly. The ISI and the PSQI global score have shown a considerably stronger association with mental health in women as compared to men. These sex differences were much smaller when sleep quality was measured by the PSQI individual question and the KSD.

### 4.3 Limitations

Our analyses were based on questionnaire data. Subjective methods have a sensitivity between 73% and 97.7%, while their specificity ranges from 50%–96% (Ibanez, Silva, & Cauli, 2018). As sleep quality is a subjective measure, it is justified to assess it using a questionnaire (Fatima, Doi, & Mamun, 2016). Furthermore, Bei *et al*. (Bei, Wiley, Allen, & Trinder, 2015) found that subjective sleep quality mediated the relationship between objective sleep and negative mood, consequently highlighting the importance of subjective sleep perception in the development of sleep related mood problems. This suggests that conclusions derived from sleep quality measures are valid. Nevertheless, the self‐report of sleep quality may be influenced by the participants’ mood and in that sense may be confounded (Krystal & Edinger, 2008).

The restricted age range of the participants in this sample is both a limitation and a strength; a strength because it helps to eliminate many confounding factors that are associated with age; a limitation because it prevents extrapolating our conclusions to the older population.

Another limitation is that the study population included more males than females. This was due to a differential response during the laboratory study recruitment phase, which generally attracts more men. However, the observed gender differences warrant further research into this area with larger samples sizes of equal proportions of men and women.

Finally the “work‐start time” may interact with chronotype in modulating sleep duration and quality and therefore could have been included as a predictor in the multivariate models. In the current analyses, however, we were interested in the predictive value of direct measures of self‐reported sleep quality, duration and chronotype and it was not our aim to analyse how other factors such as work‐start time, may interact with these direct measures.

## 5 CONCLUSION AND OUTLOOK

This study found that sleep quality is the strongest independent predictor of mental and physical health outcomes in young healthy adults, with significant effect for both sex groups but stronger association seen in women than in men. Therefore, the results of this study imply that sleep quality should be used in the assessment and treatment of mental health conditions in clinical settings, particularly for women. Further research is needed to understand the relationship between subjective sleep quality and objective sleep measures (e.g. Della Monica, Johnsen, Atzori, Groeger, & Dijk, 2018) and identify other determinants of subjective sleep quality.

## AUTHOR CONTRIBUTIONS

JAG and D‐JD designed research; D‐JD directed the research; ASL performed research; KM and ASL analyzed data; and KM, ASL, JAG, and D‐JD wrote the paper.

## Supporting information

Supplementary Material

## Data Availability

Data available on request from the authors.
