# Supplementary material for: Self‐reported sleep quality is more closely associated with mental and physical health than chronotype and sleep duration in young adults: A multi‐instrument analysis
Source: J Sleep Res. 2020 Aug 11;30(1):e13152. doi: 10.1111/jsr.13152 (PMC11475679; doi:10.1111/jsr.13152)
Supplement: Supplementary file 1 — Supplementary Material [file JSR-30-e13152-s001.docx]

**Supplementary Material**

**SUPPLEMENTARY METHODS**

**Recruitment**

Participants were recruited using posters, advertisements in local newspapers, by radio, and through Web sites. There were no restrictions to participation based on ethnicity or country of origin. Recruitment and screening took place between November 2008 and March 2010, during which a total of seven hundred and four people participated. They attended the screening phase of three laboratory-based sleep research projects where they provided written informed consent. Eligibility criteria for screening were that the Subjects were healthy, non-smokers, aged between 20 and 35 years, did not do any shift-work during the last year and had a BMI ranging between 18 and 30

**Short Description of Study Questionnaires**

**Medical Questionnaire (MQ).** The MQ is a 25-item questionnaire administered according to the Standard Operating Procedures of the Surrey Clinical Research Centre. It includes questions about general demo- graphics (age, sex, weight, and height), health-related information (past and current medical conditions, medications), habitual sleep duration and shift-work.

**British Sleep Survey (BSS).** The BSS was developed to provide representative data on British adults. Respondents are asked what their average sleep in bed was per day in the preceding week, what their average non-bed sleep was per day, and which (if any) days during the preceding week differed from this average, and what the estimates are for each of those days. Similar questions are asked with regard to times of retiring and arising. There are also questions related to demo- graphics, including ethnicity (Groeger, Zijlstra, & Dijk, 2004).

**Morningness Eveningness Questionnaires (MEQ).** The MEQ represents a widely used tool for assessing preferred timing of wake activities and sleep across 19 questions and provides a quantitative measure (total score) of diurnal preference that ranges from 16 to 86. Higher values indicate increased morning preference (Horne & Ostberg, 1976).

**Munich ChronoType Questionnaire (MCTQ).** The MCTQ asks about sleep habits in the present circumstances and addresses sleep-wake timing separately for workdays and free days. The self-assessment question assesses chronotype on a scale ranging from 0 (extreme early type) to 6 (extreme late type)(Roenneberg, Wirz-Justice, & Merrow, 2003). For all variables extracted from the MCTQ, we also computed the difference between workdays and free days (free days − workdays) and the average between workdays and free days.

**Pittsburgh Sleep Quality Index (PSQI).** The PSQI is a 24-item questionnaire that generates seven component scores (sleep quality, sleep latency, sleep duration, habitual sleep efficiency, sleep disturbances, use of sleep medications, and daytime dysfunction). The addition of these seven components yields a global score of subjective sleep quality ranging from 0 to 21, where a higher score is indicative of poorer subjective sleep quality. Here, we only report the global score. The questions refer to the last month (Buysse, Reynolds, Monk, Berman, & Kupfer, 1989).

**Karolinska Sleep Diary (KSD).** The KSD is a 10-item questionnaire assessing the last night sleep-wake timing and sleep quality. Sleep quality is assessed on a scale ranging from 1 (best ever) to 9 (worst ever) (Akerstedt, Hume, Minors, & Waterhouse, 1994).

**Insomnia Severity Index (ISI).** The ISI is a 7-item questionnaire assessing dissatisfaction with sleep during the last 2 weeks, with a maximum of 28 points. A higher score indicates more severe insomnia (Bastien, Vallieres, & Morin, 2001).

**SF-36 Health Survey (SF-36v2).** The SF-36 contains eight subscales assessing physical functioning, role physical, role emotion, vitality, mental health, social functioning, body pain, and general health in general and during the last 4 wks. For each subscale, scores are summed and transformed to generate a score ranging from a 0 to 100, with higher scores indicating better health-related quality of life. Finally, all dimensions were grouped into two summary or global dimensions: the physical health component and the mental health component. The computation of the aggregate scores consists of several steps. In the first step, a standardized (Z-score) is computed by subtracting the mean 0–100 general US population for each SF-36 scale and dividing the difference by the corresponding scale standard deviation. After the Z- score has been computed for each SF-36V2 scale, in the second step the aggregate score is computed by multiplying each SF-36 scale Z-score by its respective mental factor score coefficient and summing the eight products. In the third step, each component score was transformed to the norm-based (50, 10) scoring by multiplying each aggregate component scale score by 10 and adding the resulting product to 50. A higher score indicates better health based on the findings of the SF-36v2 Health Survey (1992–2002 by Health Assessment Lab, Medical Outcomes Trust, Hanover, Germany, and QualityMetric Incorporated, Lincoln, NE, USA) (Ware & Sherbourne, 1992).

**General Health Questionnaire (GHQ).** The GHQ is a measure of current mental health consisting of 12 items assessing whether the participant has experienced a specific symptom or behaviour during the last 4 wks. Each item is rated on a 4-point scale. The total score ranges between 0 and 36 (i.e., the lower the score the healthier the person) (D. Goldberg, 1978; L. R. Goldberg, 1990).

**Dutch Eating Behaviour Questionnaire (DEBQ).** The DEBQ is a widely used scale consisting of 33 items measuring three different psychologically based eating behaviours: (i) emotional eating (food intake as a response to an emotional factor); (ii) external eating (eating in response to food-related stimuli, regardless of the internal states of hunger and satiety); and (iii) restrained eating (effort to refrain from eating). A higher score indicates stronger presence of a certain eating behaviour (van Strien, Frijters, Bergers, & Defares, 1986).

**Big Five Inventory (BFI).** This instrument is based on the five-factor model of personality and measures openness, extraversion, agreeableness, conscientious- ness, and neuroticism. Openness refers to propensity to novelty, tolerance of different values. Extraversion refers to aspects such as activity, assertiveness, and self-confidence. Agreeableness refers to concern and sensitiveness toward others and their needs. Conscientiousness refers to self-regulation in proactive and inhibitory mode. Neuroticism refers to the inability to cope adequately with one’s own anxiety and emotionality and to control irritation and anger. A higher score indicates the stronger presence of a certain personality dimension (L. R. Goldberg, 1990; McCrae & Costa, 1987).

**Behaviour Inhibition System–Behaviour Activation System (BIS-BAS).** The questionnaire is based on the theory that a behavioural approach/activation system (BAS) regulates appetitive motives, which drive approach to something desired. A behavioural inhibition system (BIS) is believed to govern aversive motives, which drive removal from something unpleasant. The BIS-BAS, which consists of 24 items, measures individual differences in the sensitivity of these systems. The characterization is provided along four factors: BAS Drive, BAS Fun Seeking, BAS Reward Responsiveness, and BIS. The BIS comprises 7 items about anticipation of punishment. The BAS Reward Responsiveness (RR) has 5 items about anticipation or occurrence of reward. The BAS Drive has 4 items about pursuit of desired goals. The BAS fun seeking has 4 items about desire for new rewards and impulsive approach to potential rewards. Higher values indicate stronger presence of a certain motive (Carver & White, 1994).

**Positive Affect Negative Affect Scale (PANAS).** The PANAS is a reliable 20-item self-report measure of dis- positional positive and negative affect experienced during the last few weeks. The positive affect (PA) score reflects positive engagement with the environment, whereas the negative affect (NA) score reflects negative engagement with the environment. The lower ends of each dimension suggest the absence of those affective activations (Watson, Clark, & Tellegen, 1988).

**Statistical analysis – multivariate regression models**

Although the three primary health outcome measures were general psychiatric health, mental and physical health, in the first step the analyses were conducted on all self-reported measures of health, psychological well-being and personality. These multivariate models were then repeated to see whether significance was maintained when sex, age, ethnicity, work status, gross income, marital status and alcohol consumption were included in the model. This study reports analyses based on one measure for each of the sleep factors in Table 2. Chronotype was measured using the total score of the MEQ as this is one of the most widely used and most valid measures for chronotype (Taillard, Philip, Chastang, & Bioulac, 2004; Tonetti, 2007). The measure used for sleep duration was based on the average sleep period time (SPT) calculation from the MCTQ, which takes sleep latency into account and was based on both work and free days. The sleep quality measure used was the PSQI global score as it is one of the most widely used scales for sleep quality having shown sound reliability, validity, responsiveness and interpretability (Buysse et al., 1989; Devine, Hakim, & Green, 2005).

Given that sleep quality can be measured in multiple way, in a second step, the regression models were repeated the other three sleep quality measures (PSQI global score, ISI total score, the single sleep quality questions from the PSQI and the KSD referring to the last month and the last day, respectively) and the results of this are also indicated in Table 2; the complete tables of these analyses can be found in Supplementary Tables 1a, b and c.

The independent effect of chronotype, sleep duration and sleep quality on outcome measures of physical and mental health and psychological wellbeing was estimated by Local effect size calculations of Cohen’s *f*^2.^ This quantifies the proportion of variance explained by adding a sleep or chronotype predictor to the model with confounders alone (Selya, Rose, Dierker, Hedeker, & Mermelstein, 2012). The results of these analyses are included in Table 2 and supplementary Tables 1a, b and c and represented in Figure 1 (limited to the three main health outcome measures).

In order to mitigate the effect of the multivariate model chosen for the analysis, in a third step we repeated the analyses using 27 individual multivariate models for each of the three main health outcome dependent variable (general psychiatric, mental and physical health). The 27 models included all possible combination between three different outcome measures for each predictor (*chronotype* measured by the global MEQ score, the MSF and MCTQ_Iam_ from the MCTQ, *sleep quality* measured by the PSQI global score, PSQI single sleep quality question and KSD single sleep quality question and *sleep duration* measured by the BSS, PSQI and MCTQ) (See supplementary Tables 2a, b and c). The analyses were then repeated again to see whether significance was maintained when sex, age, ethnicity, work status, gross income, marital status and alcohol consumption were included in the model (same tables). Local effect sizes were also indicated for each model. We then calculated the average local effect size of each predictor across all multivariate models for each dependent measure (Figure 2).

In a next step the regression model analyses were then repeated for each sex separately (2*27 multivariate models). The results of these analyses are indicated in the supplementary Tables 2a, b and c. As to before, an average effect size of chronotype, sleep quality and sleep duration across the 27 different regression model combinations for general psychiatric, mental and physical health were calculated and plotted separately for men and women in Figure 3.

**SUPPLEMENTARY RESULTS**

**Supplementary Table 1**: Multicollinearity assessment for all regression models

| **Models** |  |  | **Chronotype** | |  |  | **Sleep quality** | |  |  | **Sleep duration** | |
| --- | --- | --- | --- | --- | --- | --- | --- | --- | --- | --- | --- | --- |
|  |  | Measures | Tolerance | VIF |  | Measures | Tolerance | VIF |  | Measures | Tolerance | VIF |
| 1 |  | MEQ | 0.950 | 1.053 |  | PSQI_g_ | 0.954 | 1.049 |  | MCTQ_SD_ | 0.982 | 1.019 |
| 2 |  | MEQ | 0.971 | 1.030 |  | PSQI_sq_ | 0.977 | 1.024 |  | MCTQ_SD_ | 0.984 | 1.016 |
| 3 |  | MEQ | 0.914 | 1.094 |  | ISI | 0.923 | 1.083 |  | MCTQ_SD_ | 0.986 | 1.015 |
| 4 |  | MEQ | 0.969 | 1.032 |  | KSD_sq_ | 0.976 | 1.025 |  | MCTQ_SD_ | 0.986 | 1.014 |
| 5 |  | MSF_CORR_ | 0.929 | 1.076 |  | PSQI_g_ | 0.955 | 1.047 |  | MCTQ_SD_ | 0.957 | 1.045 |
| 6 |  | MSF_CORR_ | 0.942 | 1.061 |  | PSQI_sq_ | 0.968 | 1.033 |  | MCTQ_SD_ | 0.961 | 1.040 |
| 7 |  | MSF_CORR_ | 0.923 | 1.083 |  | ISI | 0.951 | 1.051 |  | MCTQ_SD_ | 0.966 | 1.036 |
| 8 |  | MSF_CORR_ | 0.958 | 1.044 |  | KSD_sq_ | 0.981 | 1.019 |  | MCTQ_SD_ | 0.970 | 1.031 |
| 9 |  | MCTQ_Iam_ | 0.962 | 1.040 |  | PSQI_g_ | 0.972 | 1.029 |  | MCTQ_SD_ | 0.975 | 1.025 |
| 10 |  | MCTQ_Iam_ | 0.977 | 1.024 |  | PSQI_sq_ | 0.987 | 1.014 |  | MCTQ_SD_ | 0.980 | 1.020 |
| 11 |  | MCTQ_Iam_ | 0.951 | 1.051 |  | ISI | 0.965 | 1.036 |  | MCTQ_SD_ | 0.982 | 1.019 |
| 12 |  | MCTQ_Iam_ | 0.967 | 1.034 |  | KSD_sq_ | 0.979 | 1.021 |  | MCTQ_SD_ | 0.982 | 1.018 |
| 13 |  | MEQ | 0.942 | 1.062 |  | PSQI_g_ | 0.867 | 1.153 |  | BSS_SD_ | 0.891 | 1.123 |
| 14 |  | MEQ | 0.974 | 1.027 |  | PSQI_sq_ | 0.963 | 1.038 |  | BSS_SD_ | 0.971 | 1.030 |
| 15 |  | MEQ | 0.911 | 1.098 |  | ISI | 0.914 | 1.094 |  | BSS_SD_ | 0.974 | 1.027 |
| 16 |  | MEQ | 0.967 | 1.034 |  | KSD_sq_ | 0.961 | 1.041 |  | BSS_SD_ | 0.974 | 1.027 |
| 17 |  | MSF_CORR_ | 0.923 | 1.084 |  | PSQI_g_ | 0.859 | 1.164 |  | BSS_SD_ | 0.876 | 1.142 |
| 18 |  | MSF_CORR_ | 0.954 | 1.048 |  | PSQI_sq_ | 0.953 | 1.049 |  | BSS_SD_ | 0.962 | 1.040 |
| 19 |  | MSF_CORR_ | 0.926 | 1.080 |  | ISI | 0.935 | 1.069 |  | BSS_SD_ | 0.972 | 1.029 |
| 20 |  | MSF_CORR_ | 0.964 | 1.037 |  | KSD_sq_ | 0.969 | 1.032 |  | BSS_SD_ | 0.970 | 1.031 |
| 21 |  | MCTQ_Iam_ | 0.965 | 1.036 |  | PSQI_g_ | 0.892 | 1.121 |  | BSS_SD_ | 0.898 | 1.113 |
| 22 |  | MCTQ_Iam_ | 0.985 | 1.015 |  | PSQI_sq_ | 0.974 | 1.027 |  | BSS_SD_ | 0.973 | 1.028 |
| 23 |  | MCTQ_Iam_ | 0.955 | 1.048 |  | ISI | 0.958 | 1.044 |  | BSS_SD_ | 0.981 | 1.019 |
| 24 |  | MCTQ_Iam_ | 0.970 | 1.031 |  | KSD_sq_ | 0.967 | 1.034 |  | BSS_SD_ | 0.978 | 1.022 |
| 25 |  | MEQ | 0.936 | 1.069 |  | PSQI_g_ | 0.774 | 1.292 |  | PSQI_SD_ | 0.798 | 1.253 |
| 26 |  | MEQ | 0.977 | 1.024 |  | PSQI_sq_ | 0.956 | 1.046 |  | PSQI_SD_ | 0.966 | 1.035 |
| 27 |  | MEQ | 0.917 | 1.090 |  | ISI | 0.913 | 1.095 |  | PSQI_SD_ | 0.979 | 1.021 |
| 28 |  | MEQ | 0.972 | 1.029 |  | KSD_sq_ | 0.966 | 1.036 |  | PSQI_SD_ | 0.983 | 1.017 |
| 39 |  | MSF_CORR_ | 0.914 | 1.094 |  | PSQI_g_ | 0.767 | 1.303 |  | PSQI_SD_ | 0.781 | 1.280 |
| 30 |  | MSF_CORR_ | 0.956 | 1.046 |  | PSQI_sq_ | 0.946 | 1.057 |  | PSQI_SD_ | 0.955 | 1.047 |
| 31 |  | MSF_CORR_ | 0.933 | 1.072 |  | ISI | 0.939 | 1.065 |  | PSQI_SD_ | 0.973 | 1.028 |
| 32 |  | MSF_CORR_ | 0.966 | 1.035 |  | KSD_sq_ | 0.973 | 1.028 |  | PSQI_SD_ | 0.973 | 1.028 |
| 33 |  | MCTQ_Iam_ | 0.957 | 1.045 |  | PSQI_g_ | 0.800 | 1.250 |  | PSQI_SD_ | 0.805 | 1.243 |
| 34 |  | MCTQ_Iam_ | 0.986 | 1.015 |  | PSQI_sq_ | 0.968 | 1.033 |  | PSQI_SD_ | 0.967 | 1.034 |
| 35 |  | MCTQ_Iam_ | 0.957 | 1.045 |  | ISI | 0.958 | 1.043 |  | PSQI_SD_ | 0.981 | 1.020 |
| 36 |  | MCTQ_Iam_ | 0.972 | 1.028 |  | KSD_sq_ | 0.971 | 1.030 |  | PSQI_SD_ | 0.983 | 1.017 |

Collinearity assessment for all possible models including the various combinations of multiple measures of chronotype, sleep quality and sleep duration. MEQ- Morningness-Evenigness Questionnaire total score, MCTQ_Iam_- Munich Chronotype Questionnaire diurnal preference, MSF_corr_- Midpoint of sleep on free days corrected for corrected for the sleep debt accumulated during the week (see methods), PSQI_sq_- Pittsburgh Sleep Quality Index single question on sleep quality, PSQI_g_- Pittsburgh Sleep Quality Index global score, ISI- Insomnia Severity Index total score, KSD_sq_ – Karolinska Sleepiness Sleep quality MCTQ_SD_- Munich Chronotype Questionnaire sleep duration based on work and free days, BSS_SD_- British Sleep Survey sleep duration based on last week, PSQI_SD_- Pittsburgh Sleep Quality Index sleep duration based on the last month.

**Supplementary Table 2a:** The Independent Contribution of Chronotype, Sleep Quality (ISI) and Sleep Duration on Measures of Health and Psychological Characteristics.

| Physical and Psychological Wellbeing Variables | Chronotype | | | | |  | | Sleep Quality | | | | Sleep Duration | | | | | |  |
| --- | --- | --- | --- | --- | --- | --- | --- | --- | --- | --- | --- | --- | --- | --- | --- | --- | --- | --- |
|  | Beta | 95.0 Confidence Interval for B | p-value | p-value controlled | *f^2^* | Beta | 95.0 Confidence Interval for B | | p- value | p-value controlled | *f^2^* | | Beta | 95.0 Confidence Interval for B | p-value | p-value  controlled | *f^2^* | Adjusted  R Square  Without (with covariates) |
| Psychiatric Health  Mental Health | -0.054  0.091 | (-0.088, -0.021)  (0.020, 0.190) | **0.002**  **0.015** | **0.012**  **0.017** | 0.017  0.010 | 0.315  -0.421 | (0.270, 0.444)  (-1.490, -1.048) | | **<0.0001**  **<0.0001** | **<0.0001**  **<0.0001** | 0.106  0.207 | | 0.024  0.008 | (-0.174, 0.338)  (-0.579, 0.716) | 0.532  0.835 | 0.477  0.751 | 0.001  0.000 | 0.133 (0.156)  0.203 (0.216) |
| Physical Health  BMI | -0.019  0.025 | (-0.089, 0.052)  (-0.021, 0.039) | 0.607  0.561 | 0.389  0.944 | 0.000  0.001 | -0.931  0.032 | (-1.115, -0.746)  (-0.048, 0.108) | | **<0.0001**  0.452 | **<0.0001**  0.270 | 0.159  0.001 | | -0.097  -0.050 | (-0.638, 0.444)  (-0.369, 0.087) | 0.726  0.226 | 0.829  0.755 | 0.000  0.002 | 0.139 (0.141)  -0.001 (0.041) |
| *DEBQ*  Restrained Eating  Emotional Eating  External Eating | 0.012  -0.007  -0.017 | (0.004, 0.020)  (-0.014, 0.001)  (-0.024,-0.011) | **0.004**  0.071  **<0.0001** | 0.054  **0.034**  **<0.0001** | 0.013  0.005  0.044 | 0.041  0.037  0.020 | (0.020, 0.062)  (0.018, 0.056)  (0.003, 0.037) | | **<0.0001**  **<0.0001**  **0.020** | **0.001**  **0.001**  **0.013** | 0.024  0.009  0.009 | | 0.036  0.001  -0.015 | (-0.026, 0.097)  (-0.056, 0.058)  (-0.066, 0.035) | 0.257  0.971  0.549 | 0.949  0.420  0.429 | 0.002  0.000  0.001 | 0.025 (0.131)  0.032 (0.125)  0.060 (0.075) |
| *Big Five Inventory*  Openness  Conscientiousness  Extraversion  Agreeableness Neuroticism | -0.083  0.211  0.046  0.093  -0.064 | (-0.106, 0.000)  (0.159, 0.263)  (-0.022, 0.080)  (0.045, 0.142)  (-0.116, -0.012) | **0.050**  **<0.0001**  0.269  **<0.0001**  **0.016** | 0.082  **<0.0001**  0.093  **0.005**  **0.006** | 0.006  0.107  0.002  0.024  0.010 | -0.098  -0.233  -0.140  -0.207  0.502 | (-0.303, -0.026)  (-0.370, -0.096)  (-0.361, -0.095)  (-0.334, -0.080)  (0.367, 0.636) | | **0.020**  **0.001**  **0.001**  **0.001**  **<0.0001** | **0.036**  **<0.0001**  **<0.0001**  **<0.0001**  **<0.0001** | 0.009  0.018  0.019  0.017  0.087 | | -0.045  0.256  0.017  0.521  0.062 | (-0.636, 0.177)  (-0.147, 0.658)  (-0.310, 0.475)  (0.148, 0.894)  (-0.336, 0.459) | 0.266  0.213  0.679  **0.006**  0.761 | 0.349  0.310  0.948  **0.028**  0.922 | 0.002  0.003  0.000  0.012  0.000 | 0.008 (0.012)  0.135 (0.173)  0.021 (0.042)  0.056 (0.123)  0.104 (0.162) |
| *PANAS*  Positive Affect  Negative Affect | 0.201  -0.024 | (0.092, 0.212)  (-0.072, 0.024) | **<0.0001**  0.332 | **<0.0001**  0.286 | 0.040  0.002 | -0.152  0.582 | (-0.458, -0.144)  (0.454, 0.709) | | **<0.0001**  **<0.0001** | **<0.0001**  **<0.0001** | 0.023  0.132 | | -0.043  -0.098 | (-0.723, 0.203)  (-0.471, 0.275) | 0.271  0.607 | 0.379  0.607 | 0.002  0.000 | 0.079 (0.084)  0.129 (0.131) |
| *BIS-BAS*  BIS  BAS reward  BAS drive  BAS fun seeking | 0.033  -0.022  -0.021  0.022 | (-0.022, 0.051)  (-0.042, -0.002)  (-0.043, 0.001)  (0.001, 0.042) | 0.429  **0.029**  0.067  **0.043** | 0.124  0.051  0.084  0.794 | 0.001  0.008  0.006  0.007 | -0.167  -0.020  0.040  0.035 | (-0.290, -0.100)  (-0.071, 0.032)  (-0.017, 0.098)  (-0.032, 0.078) | | **<0.0001**  0.456  0.170  0.404 | **0.001**  0.545  0.186  0.795 | 0.027  0.001  0.003  0.001 | | -0.021  0.036  0.033  -0.001 | (-0.355, 0.207)  (-0.117, 0.189)  (-0.135, 0.201)  (-0.163, 0.160) | 0.607  0.647  0.703  0.985 | 0.924  0.660  0.741  0.877 | 0.000  0.000  0.000  0.000 | 0.028 (0.148)  0.004 (0.027)  0.007 (0.013)  0.002 (0.083) |

Regression model including chronotype, sleep quality and sleep duration as predictors and measures of physical and mental health and psychological wellbeing as dependent variables. Chronotype is measured as the total score of the Morningness-Evenigness Questionnaire (MEQ) where higher score indicates greater morning preference. Sleep quality is measured as the total score from the Insomnia Severity Index (ISI), where a higher score indicates poorer sleep quality. Sleep duration is measured as the average sleep period time across work days and free days from the Munich Chronotype Questionnaire (MCTQ) where an increasing score indicates a longer sleep duration. Higher scores of general psychiatric health indicate poorer health, whilst lower scores in physical and mental health indicate poorer health outcomes. Higher depression scores indicate greater risk of depression. For the Dutch Eating Behaviour Questionnaire (DEBQ) higher scores indicate greater endorsement of the eating behaviour, for the Big Five Inventory a higher score indicates the stronger presence of a personality trait, the Positive and Negative affect scale (PANAS) higher scores indicate greater positive or negative engagement with the environment, and for the Behavioural Inhibition and Approach systems questionnaire (BIS-BAS) lower scores indicate a greater drive through the anticipation of either punishment or reward. According to Cohen’s (1988) guidelines *f^2^*≥ 0.02, *f^2^*≥ 0.15, and *f^2^*≥ 0.35 represent small, medium, and large effect sizes, respectively. Second p value indicates significance after controlling for additional covariates: sex, age, ethnicity, work status, gross income, marital status and alcohol consumption. P values in bold indicate significant effects (p < 0.05).

**Supplementary Table 2b:** The Independent Contribution of Chronotype, Sleep Quality (PSQI single question) and Sleep Duration on Measures of Health and Psychological Characteristics.

| Physical and Psychological Wellbeing Variables | Chronotype | | | | |  | | Sleep Quality | | | | Sleep Duration | | | | | |  |
| --- | --- | --- | --- | --- | --- | --- | --- | --- | --- | --- | --- | --- | --- | --- | --- | --- | --- | --- |
|  | Beta | 95.0 Confidence Interval for B | p-value | p-value controlled | *f^2^* | Beta | 95.0 Confidence Interval for B | | p- value | p-value controlled | *f^2^* | | Beta | 95.0 Confidence Interval for B | p-value | p-value  controlled | *f^2^* | Adjusted  R Square  Without (with covariates) |
| Psychiatric Health  Mental Health | -0.077  0.201 | (-0.110,-0.044)  (0.114,0.281) | **<0.0001**  **<0.0001** | **<0.0001**  **<0.0001** | 0.034  0.033 | 1.464  -4.482 | (0.978, 1.951)  (-5.765, -3.198) | | **<0.0001**  **<0.0001** | **<0.0001**  **<0.0001** | 0.056  0.074 | | 0.077  0.064 | (-0.182,0.337)  (-0.619, 0.748) | 0.559  0.853 | 0.571  0.756 | 0.001  0.000 | 0.090 (0.112)  0.105 (0.126) |
| Physical Health  BMI | 0.043  0.005 | (-0.028, 0.114)  (-0.024, 0.034) | 0.233  0.738 | 0.443  0.729 | 0.002  0.000 | -3.172  0.007 | (-4.222, -2.123)  (-0.417, 0.431) | | **<0.0001**  0.974 | **<0.0001**  0.960 | 0.055  0.000 | | -0.106  -0.176 | (-0.665, 0.453)  (-0.402, 0.049) | 0.710  0.125 | 0.717  0.591 | 0.000  0.004 | 0.054 (0.071)  -0.001 (0.042) |
| *DEBQ*  Restrained Eating  Emotional Eating  External Eating | 0.007  -0.010  -0.019 | (-0.001, 0.015)  (-0.017,-0.003)  (-0.025, -0.013) | 0.067  **0.006**  **<0.0001** | 0.367  **0.003**  **<0.0001** | 0.005  0.012  0.055 | 0.067  0.109  0.044 | (-0.050, 0.184)  (0.004, 0.214)  (-0.050, 0.137) | | 0.261  **0.041**  0.362 | 0.134  **0.021**  0.307 | 0.002  0.007  0.001 | | 0.024  -0.003  -0.011 | (-0.038, 0.086)  (-0.060, 0.053)  (-0.061, 0.039) | 0.455  0.904  0.665 | 0.758  0.327  0.518 | 0.001  0.000  0.000 | 0.002 (0.116)  0.017 (0.113)  0.052 (0.061) |
| *Big Five Inventory*  Openness  Conscientiousness  Extraversion  Agreeableness Neuroticism | -0.037  0.233  0.049  0.107  -0.100 | (-0.088, 0.014)  (0.183, 0.283)  (0.000, 0.098)  (0.062, 0.153)  (-0.151, -0.050) | 0.157  **<0.0001**  0.050  **<0.0001**  **<0.0001** | 0.162  **<0.0001**  **0.011**  **<0.0001**  **<0.0001** | 0.003  0.134  0.006  0.034  0.024 | -0.833  -0.402  -0.948  -1.419  2.451 | (-1.594, -0.720)  (-1.156, 0.352)  (-1.676, -0.219)  (-2.102, -0.736)  (1.704, 3.198) | | **0.032**  0.296  **0.011**  **<0.0001 <0.0001** | 0.021  0.208  **0.005**  **<0.0001**  **<0.0001** | 0.007  0.002  0.010  0.027  0.066 | | -0.195  0.236  0.102  0.489  0.100 | (-0.601, 0.211)  (-0.168, 0.640)  (-0.287, 0.491)  (0.125, 0.853)  (-0.300, 0.500) | 0.346  0.252  0.607  **0.009**  0.632 | 0.369  0.321  0.902  **0.040**  0.786 | 0.001  0.002  0.000  0.011  0.000 | 0.005 (0.012)  0.121 (0.160)  0.014 (0.034)  0.069 (0.139)  0.090 (0.157) |
| *PANAS*  Positive Affect  Negative Affect | 0.163  -0.067 | (0.105, 0.222)  (-0.116, -0.019) | **<0.0001**  **0.006** | **<0.0001**  **0.007** | 0.048  0.012 | -1.333  1.875 | (-2.203,-0.462)  (1.162, 2.589) | | **0.003**  **<0.0001** | **<0.0001**  **<0.0001** | 0.014  0.043 | | -0.289  -0.091 | (-0.754, 0.176)  (-0.475, 0.293) | 0.223  0.642 | 0.293  0.466 | 0.002  0.000 | 0.066 (0.076)  0.054 (0.065) |
| *BIS-BAS*  BIS  BAS reward  BAS drive  BAS fun seeking | 0.029  -0.017  -0.020  0.025 | (-0.006, 0.064)  (-0.036, 0.002)  (-0.041, 0.001)  (0.005, 0.045) | 0.102  0.072  0.059  **0.016** | **0.019**  0.118  **0.046**  0.597 | 0.004  0.005  0.006  0.009 | -0.794  0.108  0.315  0.298 | (-1.313, -0.276)  (-0.173, 0.390)  (0.000, 0.630)  (0.002, 0.593) | | **0.003**  0.450  **0.050**  **0.049** | **0.002**  0.333  **0.036**  **0.049** | 0.014  0.001  0.006  0.006 | | -0.057  0.029  0.044  0.007 | (-0.335, 0.220)  (-0.122, 0.180)  (-0.123, 0.211)  (-0.152, 0.166) | 0.685  0.710  0.605  0.928 | 0.990  0.673  0.697  0.733 | 0.000  0.000  0.000  0.000 | 0.016 (0.140)  0.002 (0.029)  0.009 (0.020)  0.009 (0.084) |

Regression model including chronotype, sleep quality and sleep duration as predictors and measures of physical and mental health and psychological wellbeing as dependent variables. Chronotype is measured as the total score of the Morningness-Evenigness Questionnaire (MEQ) where higher score indicates greater morning preference. Sleep quality is measured as the single sleep quality question from the Pittsburgh Sleep Quality Index (PSQI), where a higher score indicates poorer sleep quality. Sleep duration is measured as the average sleep period time across work days and free days from the Munich Chronotype Questionnaire (MCTQ) where an increasing score indicates a longer sleep duration. Higher scores of general psychiatric health indicate poorer health, whilst lower scores in physical and mental health indicate poorer health outcomes. Higher depression scores indicate greater risk of depression. For the Dutch Eating Behaviour Questionnaire (DEBQ ) higher scores indicate greater endorsement of the eating behaviour, for the Big Five Inventory a higher score indicates the stronger presence of a personality trait, the Positive and negative affect scale (PANAS) higher scores indicate greater positive or negative engagement with the environment, and for the Behavioural Inhibition and Approach systems questionnaire (BIS-BAS) lower scores indicate a greater drive through the anticipation of either punishment or reward. According to Cohen’s (1988) guidelines *f^2^*≥ 0.02, *f^2^*≥ 0.15, and *f^2^*≥ 0.35 represent small, medium, and large effect sizes, respectively. Second p value indicates significance after controlling for additional covariates: sex, age, ethnicity, work status, gross income, marital status and alcohol consumption. P values in bold indicate significant effects (p < 0.05).

**Supplementary Table 2c:** The Independent Contribution of Chronotype, Sleep Quality (KSD) and Sleep Duration on Measures of Health and Psychological Characteristics.

| Physical and Psychological Wellbeing Variables | Chronotype | | | | | Sleep Quality | | | | | Sleep Duration | | | | |  |
| --- | --- | --- | --- | --- | --- | --- | --- | --- | --- | --- | --- | --- | --- | --- | --- | --- |
|  | Beta | 95.0 Confidence Interval for B | p-value | p-value controlled | *f^2^* | Beta | 95.0 Confidence Interval for B | p- value | p-value controlled | *f^2^* | Beta | 95.0 Confidence Interval for B | p-value | p-value  controlled | *f^2^* | Adjusted  R Square  Without (with covariates) |
| Psychiatric Health  Mental Health | -0.073  0.191 | (-0.106, -0.040)  (0.104, 0.278) | **<0.0001**  **<0.0001** | **0.0001**  **<0.0001** | 0.026  0.013 | 0.608  -1.801 | (0.417, 0.800)  (-2.308, -1.293) | **<0.0001**  **<0.0001** | **<0.0001**  **<0.0001** | 0.061  0.030 | 0.065  0.119 | (-0.195, 0.325)  (-0.568, 0.807) | 0.623  0.733 | 0.764  0.507 | 0.000  0.000 | 0.095 (0.107)  0.106 (0.125) |
| Physical Health  BMI | 0.053  0.004 | (-0.019, 0.124)  (-0.025, 0.033) | 0.148  0.780 | 0.337  0.735 | 0.006  0.001 | -0.951  -0.059 | (-1.368, -0.533)  (-0.227, 0.109) | **<0.0001**  0.491 | **<0.0001**  0.366 | 0.003  0.000 | -0.039  -0.192 | (-0.605, 0.526)  (-0.419, 0.035) | 0.891  0.097 | 0.531  0.473 | 0.009  0.010 | 0.033 (0.056)  0.001 (0.045) |
| *DEBQ*  Restrained Eating  Emotional Eating  External Eating | 0.006  -0.010  -0.018 | (-0.001, 0.014)  (-0.017, -0.002)  (-0.025, -0.012) | 0.107  **0.009**  **<0.0001** | 0.452  **0.006**  **<0.0001** | 0.001  0.001  0.002 | -0.017  0.020  0.037 | (-0.063, 0.030)  (-0.021, 0.061)  (0.000, 0.074) | 0.483  0.344  0.051 | 0.861  0.105  0.065 | 0.000  0.010  0.003 | 0.013  -0.006  -0.011 | (-0.050, 0.075)  (-0.062, 0.051)  (-0.061, 0.039) | 0.689  0.836  0.676 | 0.519  0.270  0.488 | 0.004  0.004  0.001 | 0.001 (0.112)  0.009 (0.102)  0.056 (0.067) |
| *Big Five Inventory*  Openness  Conscientiousness  Extraversion  Agreeableness Neuroticism | -0.031  0.216  0.055  0.097  -0.103 | (-0.082, 0.021)  (0.167, 0.266)  (0.005, 0.104) (0.051,0.142)  (-0.155, -0.051) | 0.241  **<0.0001**  **0.030**  **<0.0001**  **<0.0001** | 0.238  **<0.0001**  **0.007**  **0.001**  **<0.0001** | 0.006  0.002  0.000  0.018  0.000 | -0.064  -0.808  -0.291  -0.808  0.679 | (-0.367, 0.239)  (-1.101, -0.515)  (-0.580, -0.002)  (-1.073, -0.543)  (0.379, 0.978) | 0.678  **<0.0001**  **0.048**  **<0.0001**  **<0.0001** | 0.493  **<0.0001**  **0.014**  **<0.0001**  **<0.0001** | 0.001  0.001  0.097  0.005  0.014 | -0.187  0.203  0.122  0.497  0.029 | (-0.598, 0.223)  (-0.196, 0.602)  (-0.270, 0.514)  (0.136, 0.859)  (-0.381, 0.439) | 0.371  0.318  0.542  **0.007**  0.890 | 0.403  0.334  0.792  0.028  0.815 | 0.009  0.001  0.037  0.013  0.010 | -0.002 (0.006)  0.159 (0.188)  0.012 (0.032)  0.095 (0.149)  0.058 (0.114) |
| *PANAS*  Positive Affect  Negative Affect | 0.158  -0.071 | (0.100, 0.215)  (-0.120, -0.023) | **<0.0001**  **0.004** | **<0.0001**  **0.008** | 0.010  0.000 | -0.816  0.454 | (-1.156, -0.476)  (0.169, 0.739) | **<0.0001**  **0.002** | **<0.0001**  **<0.0001** | 0.016  0.000 | -0.294  -0.134 | (-0.756, 0.168)  (-0.522, 0.254) | 0.212  0.498 | 0.319  0.297 | 0.009  0.020 | 0.087 (0.090)  0.029 (0.052) |
| *BIS-BAS*  BIS  BAS reward  BAS drive  BAS fun seeking | 0.033  -0.016  -0.020  0.021 | (-0.002, 0.068)  (-0.035, 0.003)  (-0.041, 0.000)  (0.001, 0.041) | 0.067  0.098  0.055  **0.040** | **0.018**  0.157  **0.043**  0.716 | 0.000  0.002  0.003  0.004 | -0.105  0.154  0.202  0.001 | (-0.312, 0.101)  (0.044, 0.264)  (0.080, 0.325)  (-0.116, 0.119) | 0.316  **0.006**  **0.001**  0.982 | 0.174  **0.003**  **0.000**  0.291 | 0.000  0.007  0.000  0.015 | -0.027  0.048  0.060  0.005 | (-0.308, 0.253)  (-0.103, 0.198)  (-0.106, 0.226)  (-0.154, 0.165) | 0.848  0.532  0.475  0.946 | 0.790  0.539  0.583  0.715 | 0.001  0.001  0.002  0.001 | 0.003 (0.121)  0.015 (0.044)  0.022 (0.037)  0.002 (0.078) |

Regression model including chronotype, sleep quality and sleep duration as predictors and measures of physical and mental health and psychological wellbeing as dependent variables. Chronotype is measured as the total score of the Morningness-Evenigness Questionnaire (MEQ) where higher score indicates greater morning preference. Sleep quality is measured from the Karolinska Sleep Diary (KSD) single sleep quality question, where a higher score indicates poorer sleep quality. Sleep duration is measured as the average sleep period time across work days and free days from the Munich Chronotype Questionnaire (MCTQ) where an increasing score indicates a longer sleep duration. Higher scores of general psychiatric health indicate poorer health, whilst lower scores in physical and mental health indicate poorer health outcomes. Higher depression scores indicate greater risk of depression. For the Dutch Eating Behaviour Questionnaire (DEBQ ) higher scores indicate greater endorsement of the eating behaviour, for the Big Five Inventory a higher score indicates the stronger presence of a personality trait, the Positive and negative affect scale (PANAS) higher scores indicate greater positive or negative engagement with the environment, and for the Behavioural Inhibition and Approach systems questionnaire (BIS-BAS) lower scores indicate a greater drive through the anticipation of either punishment or reward. Second p value indicates significance after controlling for additional covariates: sex, age, ethnicity, work status, gross income, marital status and alcohol consumption. P values in bold indicate significant effects (p < 0.05).

**Supplementary Table 3a:** The independent contribution of Chronotype, Sleep quality and Sleep duration on general psychiatric health across all models of different predictor combinations

| **Chronotype** | | | | | | **Sleep quality** | | | | | | **Sleep duration** | | | | | | Model R^2^ without (with covariates) |
| --- | --- | --- | --- | --- | --- | --- | --- | --- | --- | --- | --- | --- | --- | --- | --- | --- | --- | --- |
| Used measure | β | 95% CI | p value | p-value  controlled | *f^2^* | Used  measure | β | 95% CI | p-value  controlled | p-value  controlled | *f^2^* | Used measure | β | 95% CI | p-value | p-value  controlled | *f^2^* |  |
| MEQ | -0.08 | (-0.11, -0.04) | **<0.0001**^M^ | **<0.0001** | 0.03 | PSQI_sq_ | 1.46 | (0.98, 1.95) | **<0.0001**^M<W^ | **<0.0001** | 0.06 | MCTQ_SD_ | 0.08 | (-0.18, 0.34) | 0.559 | 0.571 | 0.00 | 0.09 (0.11) |
| MEQ | -0.08 | (-0.11, -0.04) | **<0.0001**^M^ | **<0.0001** | 0.03 | PSQI_sq_ | 1.62 | (1.13, 2.11) | **<0.0001**^M<W^ | **<0.0001** | 0.07 | BSS_SD_ | 0.20 | (-0.13, 0.52) | 0.237 | 0.398 | 0.00 | 0.10 (0.12) |
| MEQ | -0.07 | (-0.10, -0.04) | **<0.0001**^M^ | **<0.0001** | 0.03 | PSQI_sq_ | 1.65 | (1.16, 2.13) | **<0.0001**^M<W^ | **<0.0001** | 0.07 | PSQI_SD_ | 0.32 | (0.05, 0.60) | **0.021** | **0.026** | 0.01 | 0.10 (0.12) |
| MEQ | -0.05 | (-0.09, -0.02) | **0.002**^M^ | **0.012** | 0.02 | ISI | 0.36 | (0.27, 0.44) | **<0.0001**^M<W^ | **<0.0001** | 0.11 | MCTQ_SD_ | 0.08 | (-0.17, 0.34) | 0.532 | 0.477 | 0.00 | 0.13 (0.15) |
| MEQ | -0.05 | (-0.08, -0.02) | **0.003**^M^ | **0.012** | 0.01 | ISI | 0.36 | (0.28, 0.45) | **<0.0001**^M<W^ | **<0.0001** | 0.11 | BSS_SD_ | 0.12 | (-0.20, 0.44) | 0.457 | 0.735 | 0.00 | 0.14 (0.16) |
| MEQ | -0.05 | (-0.08, -0.02) | **0.003**^M^ | **0.017** | 0.01 | ISI | 0.37 | (0.28, 0.45) | **<0.0001**^M<W^ | **<0.0001** | 0.12 | PSQI_SD_ | 0.32 | (0.05, 0.59) | **0.019** | **0.019** | 0.01 | 0.14 (0.16) |
| MEQ | -0.08 | (-0.11, -0.04) | **<0.0001**^M^ | **<0.0001** | 0.03 | PSQI_g_ | 0.46 | (0.30, 0.61) | **<0.0001**^M<W^ | **<0.0001** | 0.06 | MCTQ_SD_ | 0.12 | (-0.15, 0.38) | 0.398 | 0.390 | 0.00 | 0.10 (0.11) |
| MEQ | -0.08 | (-0.11, -0.04) | **<0.0001**^M^ | **<0.0001** | 0.03 | PSQI_g_ | 0.50 | (0.34, 0.66) | **<0.0001**^M<W^ | **<0.0001** | 0.07 | BSS_SD_ | 0.28 | (-0.07, 0.63) | 0.116 | 0.246 | 0.00 | 0.10 (0.11) |
| MEQ | -0.07 | (-0.10, -0.03) | **0.0002**^M^ | **<0.0001** | 0.02 | PSQI_g_ | 0.63 | (0.46, 0.79) | **<0.0001**^M<W^ | **<0.0001** | 0.09 | PSQI_SD_ | 0.69 | (0.38, 1.01) | **<0.0001**^M<W^ | **<0.0001** | 0.03 | 0.13 (0.14) |
| MCTQ_Iam_ | 0.36 | (0.19, 0.53) | **<0.0001**^M^ | **<0.0001** | 0.03 | PSQI_sq_ | 1.51 | (1.03, 1.99) | **<0.0001**^M<W^ | **<0.0001** | 0.06 | MCTQ_SD_ | 0.09 | (-0.17, 0.35) | 0.504 | 0.682 | 0.00 | 0.08 (0.11) |
| MCTQ_Iam_ | 0.35 | (0.18, 0.52) | **<0.0001**^M^ | **<0.0001** | 0.03 | PSQI_sq_ | 1.67 | (1.19, 2.15) | **<0.0001**^M<W^ | **<0.0001** | 0.07 | BSS_SD_ | 0.26 | (-0.06, 0.59) | 0.107 | 0.249 | 0.00 | 0.09 (0.11) |
| MCTQ_Iam_ | 0.33 | (0.17, 0.50) | **<0.0001**^M^ | **<0.0001** | 0.02 | PSQI_sq_ | 1.69 | (1.21, 2.16) | **<0.0001**^M<W^ | **<0.0001** | 0.07 | PSQI_SD_ | 0.34 | (0.07, 0.62) | **0.014** | **0.020** | 0.01 | 0.10 (0.12) |
| MCTQ_Iam_ | 0.28 | (0.11, 0.45) | **0.001**^M^ | **0.005** | 0.02 | ISI | 0.37 | (0.29, 0.46) | **<0.0001**^M<W^ | **<0.0001** | 0.12 | MCTQ_SD_ | 0.09 | (-0.16, 0.35) | 0.466 | 0.561 | 0.00 | 0.14 (0.16) |
| MCTQ_Iam_ | 0.26 | (0.09, 0.43) | **0.002**^M^ | **0.010** | 0.01 | ISI | 0.38 | (0.29, 0.46) | **<0.0001**^M<W^ | **<0.0001** | 0.13 | BSS_SD_ | 0.16 | (-0.15, 0.48) | 0.307 | 0.564 | 0.00 | 0.14 (0.16) |
| MCTQ_Iam_ | 0.25 | (0.09, 0.42) | **0.003**^M^ | **0.012** | 0.01 | ISI | 0.38 | (0.30, 0.46) | **<0.0001**^M<W^ | **<0.0001** | 0.13 | PSQI_SD_ | 0.32 | (0.05, 0.58) | **0.021** | **0.017** | 0.01 | 0.14 (0.17) |
| MCTQ_Iam_ | 0.36 | (0.18, 0.54) | **<0.0001**^M^ | **<0.0001** | 0.03 | PSQI_g_ | 0.48 | (0.32, 0.63) | **<0.0001**^M<W^ | **<0.0001** | 0.06 | MCTQ_SD_ | 0.13 | (-0.14, 0.40) | 0.335 | 0.454 | 0.00 | 0.09 (0.10) |
| MCTQ_Iam_ | 0.34 | (0.16, 0.52) | **<0.0001**^M^ | **<0.0001** | 0.02 | PSQI_g_ | 0.52 | (0.37, 0.68) | **<0.0001**^M<W^ | **<0.0001** | 0.07 | BSS_SD_ | 0.32 | (-0.02, 0.67) | 0.066 | 0.182 | 0.01 | 0.10 (0.10) |
| MCTQ_Iam_ | 0.29 | (0.12, 0.47) | **0.001**^M^ | **0.003** | 0.02 | PSQI_g_ | 0.64 | (0.48, 0.80) | **<0.0001**^M<W^ | **<0.0001** | 0.10 | PSQI_SD_ | 0.69 | (0.37, 1.00) | **<0.0001**^M≃W^ | **<0.0001** | 0.03 | 0.12 (0.13) |
| MSF_corr_ | 0.17 | (-0.07, 0.41) | 0.168 | 0.673 | 0.00 | PSQI_sq_ | 1.54 | (1.05, 2.03) | **<0.0001**^M<W^ | **<0.0001** | 0.06 | MCTQ_SD_ | 0.12 | (-0.15, 0.38) | 0.390 | 0.488 | 0.00 | 0.06 (0.08) |
| MSF_corr_ | 0.15 | (-0.09, 0.39) | 0.220 | 0.702 | 0.00 | PSQI_sq_ | 1.70 | (1.20, 2.19) | **<0.0001**^M<W^ | **<0.0001** | 0.07 | BSS_SD_ | 0.27 | (-0.06, 0.60) | 0.106 | 0.255 | 0.00 | 0.07 (0.09) |
| MSF_corr_ | 0.11 | (-0.12, 0.35) | 0.342 | 0.961 | 0.00 | PSQI_sq_ | 1.72 | (1.24, 2.20) | **<0.0001**^M<W^ | **<0.0001** | 0.07 | PSQI_SD_ | 0.36 | (0.08, 0.64) | **0.011** | **0.017** | 0.01 | 0.07 (0.10) |
| MSF_corr_ | 0.08 | (-0.16, 0.31) | 0.536 | 0.682 | 0.00 | ISI | 0.39 | (0.31, 0.48) | **<0.0001**^M<W^ | **<0.0001** | 0.13 | MCTQ_SD_ | 0.12 | (-0.13, 0.38) | 0.343 | 0.391 | 0.00 | 0.12 (0.15) |
| MSF_corr_ | 0.03 | (-0.21, 0.27) | 0.794 | 0.589 | 0.00 | ISI | 0.40 | (0.32, 0.48) | **<0.0001**^M<W^ | **<0.0001** | 0.14 | BSS_SD_ | 0.18 | (-0.13, 0.50) | 0.259 | 0.524 | 0.00 | 0.13 (0.15) |
| MSF_corr_ | 0.01 | (-0.22, 0.25) | 0.908 | 0.405 | 0.00 | ISI | 0.40 | (0.32, 0.49) | **<0.0001**^M<W^ | **<0.0001** | 0.14 | PSQI_SD_ | 0.35 | (0.08, 0.62) | **0.010** | **0.010** | 0.01 | 0.13 (0.16) |
| MSF_corr_ | 0.15 | (-0.11, 0.41) | 0.253 | 0.755 | 0.00 | PSQI_g_ | 0.51 | (0.35, 0.66) | **<0.0001**^M<W^ | **<0.0001** | 0.07 | MCTQ_SD_ | 0.17 | (-0.10, 0.44) | 0.224 | 0.295 | 0.00 | 0.07 (0.08) |
| MSF_corr_ | 0.10 | (-0.16, 0.35) | 0.457 | 0.875 | 0.00 | PSQI_g_ | 0.55 | (0.40, 0.71) | **<0.0001**^M<W^ | **<0.0001** | 0.08 | BSS_SD_ | 0.36 | (0.01, 0.72) | **0.042** | 0.136 | 0.01 | 0.08 (0.09) |
| MSF_corr_ | 0.01 | (-0.25, 0.26) | 0.962 | 0.628 | 0.00 | PSQI_g_ | 0.69 | (0.53, 0.86) | **<0.0001**^M<W^ | **<0.0001** | 0.11 | PSQI_SD_ | 0.77 | (0.45, 1.09) | **<0.0001** | **<0.0001** | 0.04 | 0.10 (0.12) |

Regression models including the combinations of multiple outcome measures of chronotype, sleep quality and sleep duration as predictors and general psychiatric health as dependent variable. Second p value indicates significance after controlling for additional covariates: sex, age, ethnicity, work status, gross income, marital status and alcohol consumption. P values in bold indicate significant effects (p < 0.05). Superscript letters indicate if p-values were significant when data for ^M^men and ^W^women were analysed separately, with </>/≃ signs to indicate which sex group had a greater/similar effect size. MEQ- Morningness-Evenigness Questionnaire total score, MCTQ_Iam_- Munich Chronotype Questionnaire diurnal preference, MSF_corr_- Midpoint of sleep on free days corrected for corrected for the sleep debt accumulated during the week (see methods), PSQI_sq_- Pittsburgh Sleep Quality Index single question, PSQI_g_- Pittsburgh Sleep Quality Index global score, ISI- Insomnia Severity Index total score, MCTQ_SD_- Munich Chronotype Questionnaire based on work and free days, BSS_SD_- British Sleep Survey sleep duration based on last week, PSQI_SD_- Pittsburgh Sleep Quality Index sleep duration based on the last month. According to Cohen’s (1988) guidelines *f^2^*≥ 0.02, *f^2^*≥ 0.15, and *f^2^*≥ 0.35 represent small, medium, and large effect sizes, respectively.

**Supplementary Table 3b**: The independent contribution of Chronotype, Sleep quality and Sleep duration on **mental health** across all models of different predictor combinations

| **Chronotype** | | | | | | **Sleep quality** | | | | | | **Sleep duration** | | | | | | Model R^2^ without (with  covariates) |
| --- | --- | --- | --- | --- | --- | --- | --- | --- | --- | --- | --- | --- | --- | --- | --- | --- | --- | --- |
| Used measure | β | 95% CI | p value | p-value  controlled | *f^2^* | Used measure | β | 95% CI | p-value | p-value  controlled | *f^2^* | Used  measure | β | 95% CI | p-value | p-value  controlled | *f^2^* |  |
| MEQ | 0.20 | (0.11, 0.29) | **<0.0001**^M<W^ | **<0.0001** | 0.03 | PSQI_sq_ | -4.48 | (-5.77, -3.20) | **<0.0001**^M<W^ | **<0.0001** | 0.07 | MCTQ_SD_ | 0.06 | (-0.62, 0.75) | 0.853 | 0.756 | 0.00 | 0.10(0.13) |
| MEQ | 0.19 | (0.10, 0.28) | **<0.0001**^M<W^ | **<0.0001** | 0.03 | PSQI_sq_ | -4.87 | (-6.17, -3.56) | **<0.0001**^M<W^ | **<0.0001** | 0.08 | BSS_SD_ | -0.19 | (-1.06, 0.68) | 0.675 | 0.760 | 0.00 | 0.11(0.14) |
| MEQ | 0.20 | (0.11, 0.28) | **<0.0001**^M<W^ | **<0.0001** | 0.03 | PSQI_sq_ | -4.93 | (-6.22, -3.65) | **<0.0001**^M<W^ | **<0.0001** | 0.09 | PSQI_SD_ | -0.13 | (-0.87, 0.60) | 0.718 | 0.797 | 0.00 | 0.11(0.14) |
| MEQ | 0.11 | (0.02, 0.19) | **0.015**^W^ | **0.017** | 0.01 | ISI | -1.27 | (-1.49, -1.05) | **<0.0001**^M<W^ | **<0.0001** | 0.21 | MCTQ_SD_ | 0.07 | (-0.58, 0.72) | 0.835 | 0.751 | 0.00 | 0.20(0.22) |
| MEQ | 0.08 | (0.00, 0.17) | 0.060^W^ | **0.041** | 0.01 | ISI | -1.32 | (-1.54, -1.11) | **<0.0001**^M<W^ | **<0.0001** | 0.23 | BSS_SD_ | -0.10 | (-0.91, 0.72) | 0.815 | 0.861 | 0.00 | 0.22 (0.23) |
| MEQ | 0.10 | (0.01, 0.18) | **0.024**^W^ | **0.031** | 0.01 | ISI | -1.33 | (-1.54, -1.11) | **<0.0001**^M<W^ | **<0.0001** | 0.23 | PSQI_SD_ | -0.23 | (-0.92, 0.45) | 0.503 | 0.645 | 0.00 | 0.22 (0.23) |
| MEQ | 0.17 | (0.08, 0.26) | **<0.0001**^M<W^ | **<0.0001** | 0.02 | PSQI_g_ | -1.88 | (-2.27, -1.49) | **<0.0001**^M<W^ | **<0.0001** | 0.15 | MCTQ_SD_ | -0.07 | (-0.75, 0.61) | 0.837 | 0.825 | 0.00 | 0.17 (0.18) |
| MEQ | 0.16 | (0.07, 0.25) | **<0.0001**^<W^ | **<0.0001** | 0.02 | PSQI_g_ | -2.05 | (-2.44, -1.65) | **<0.0001**^M<W^ | **<0.0001** | 0.17 | BSS_SD_ | -0.63 | (-1.53, 0.26) | 0.164 | 0.132 | 0.00 | 0.18 (0.19) |
| MEQ | 0.14 | (0.06, 0.23) | **0.002**^W^ | **<0.0001** | 0.02 | PSQI_g_ | -2.31 | (-2.74, -1.89) | **<0.0001**^M<W^ | **<0.0001** | 0.19 | PSQI_SD_ | -1.42 | (-2.23, -0.61) | **<0.0001**^W^ | **<0.0001** | 0.02 | 0.20 (0.21) |
| MCTQ_Iam_ | -0.80 | (-1.25, -0.35) | **<0.0001**^M<W^ | **<0.0001** | 0.02 | PSQI_sq_ | -4.78 | (-6.05, -3.51) | **<0.0001**^M<W^ | **<0.0001** | 0.08 | MCTQ_SD_ | 0.08 | (-0.60, 0.77) | 0.807 | 0.656 | 0.00 | 0.10 (0.12) |
| MCTQ_Iam_ | -0.76 | (-1.21, -0.32) | **<0.0001**^W^ | **<0.0001** | 0.02 | PSQI_sq_ | -5.16 | (-6.45, -3.87) | **<0.0001**^M<W^ | **<0.0001** | 0.10 | BSS_SD_ | -0.42 | (-1.28, 0.44) | 0.338 | 0.492 | 0.00 | 0.10 (0.13) |
| MCTQ_Iam_ | -0.75 | (-1.19, -0.31) | **<0.0001**^W^ | **<0.0001** | 0.02 | PSQI_sq_ | -5.26 | (-6.53, -3.99) | **<0.0001**^M<W^ | **<0.0001** | 0.10 | PSQI_SD_ | -0.27 | (-1.00, 0.47) | 0.475 | 0.680 | 0.00 | 0.11 (0.13) |
| MCTQ_Iam_ | -0.48 | (-0.91, -0.05) | **0.029**^W^ | **0.027** | 0.01 | ISI | -1.32 | (-1.54, -1.11) | **<0.0001**^M<W^ | **<0.0001** | 0.24 | MCTQ_SD_ | 0.12 | (-0.53, 0.76) | 0.720 | 0.672 | 0.00 | 0.21 (0.22) |
| MCTQ_Iam_ | -0.38 | (-0.81, 0.04) | 0.078^W^ | 0.057 | 0.00 | ISI | -1.37 | (-1.58, -1.16) | **<0.0001**^M<W^ | **<0.0001** | 0.26 | BSS_SD_ | -0.21 | (-1.01, 0.58) | 0.601 | 0.921 | 0.00 | 0.22 (0.23) |
| MCTQ_Iam_ | -0.41 | (-0.83, 0.02) | 0.060^W^ | 0.060 | 0.01 | ISI | -1.38 | (-1.59, -1.17) | **<0.0001**^M<W^ | **<0.0001** | 0.26 | PSQI_SD_ | -0.29 | (-0.98, 0.39) | 0.405 | 0.580 | 0.00 | 0.22 (0.23) |
| MCTQ_Iam_ | -0.63 | (-1.09, -0.17) | **0.007**^W^ | **0.005** | 0.01 | PSQI_g_ | -1.94 | (-2.33, -1.55) | **<0.0001**^M<W^ | **<0.0001** | 0.16 | MCTQ_SD_ | -0.04 | (-0.72, 0.65) | 0.914 | 0.896 | 0.00 | 0.16 (0.17) |
| MCTQ_Iam_ | -0.57 | (-1.02, -0.12) | **0.014**^W^ | **0.009** | 0.01 | PSQI_g_ | -2.11 | (-2.51, -1.72) | **<0.0001**^M<W^ | **<0.0001** | 0.18 | BSS_SD_ | -0.76 | (-1.65, 0.13) | 0.093 | 0.092 | 0.00 | 0.17 (0.18) |
| MCTQ_Iam_ | -0.48 | (-0.93, -0.03) | **0.037**^W^ | **0.022** | 0.01 | PSQI_g_ | -2.38 | (-2.80, -1.96) | **<0.0001**^M<W^ | **<0.0001** | 0.20 | PSQI_SD_ | -1.50 | (-2.30, -0.69) | **<0.0001** | **<0.0001** | 0.02 | 0.19 (0.20) |
| MSF_corr_ | -0.41 | (-1.04, 0.23) | 0.207^W^ | 0.110 | 0.00 | PSQI_sq_ | -4.84 | (-6.13, -3.56) | **<0.0001**^M<W^ | **<0.0001** | 0.08 | MCTQ_SD_ | 0.02 | (-0.67, 0.72) | 0.946 | 0.741 | 0.00 | 0.08 (0.11) |
| MSF_corr_ | -0.39 | (-1.02, 0.25) | 0.231^W^ | 0.156 | 0.00 | PSQI_sq_ | -5.23 | (-6.54, -3.92) | **<0.0001**^M<W^ | **<0.0001** | 0.09 | BSS_SD_ | -0.41 | (-1.28, 0.46) | 0.354 | 0.526 | 0.00 | 0.09 (0.12) |
| MSF_corr_ | -0.38 | (-1.01, 0.24) | 0.231^W^ | 0.151 | 0.00 | PSQI_sq_ | -5.29 | (-6.58, -4.01) | **<0.0001**^M<W^ | **<0.0001** | 0.10 | PSQI_SD_ | -0.29 | (-1.03, 0.45) | 0.437 | 0.689 | 0.00 | 0.09 (0.12) |
| MSF_corr_ | -0.04 | (-0.64, 0.56) | 0.903 | 0.559 | 0.00 | ISI | -1.36 | (-1.57, -1.14) | **<0.0001**^M<W^ | **<0.0001** | 0.25 | MCTQ_SD_ | 0.05 | (-0.60, 0.70) | 0.873 | 0.743 | 0.00 | 0.20 (0.22) |
| MSF_corr_ | 0.10 | (-0.49, 0.70) | 0.738 | 0.875 | 0.00 | ISI | -1.41 | (-1.62, -1.20) | **<0.0001**^M<W^ | **<0.0001** | 0.27 | BSS_SD_ | -0.24 | (-1.04, 0.55) | 0.549 | 0.916 | 0.00 | 0.22 (0.23) |
| MSF_corr_ | 0.06 | (-0.53, 0.65) | 0.844 | 0.832 | 0.00 | ISI | -1.42 | (-1.63, -1.21) | **<0.0001**^M<W^ | **<0.0001** | 0.27 | PSQI_SD_ | -0.36 | (-1.05, 0.32) | 0.301 | 0.527 | 0.00 | 0.22 (0.23) |
| MSF_corr_ | -0.08 | (-0.74, 0.58) | 0.809 | 0.470 | 0.00 | PSQI_g_ | -2.01 | (-2.40, -1.62) | **<0.0001**^M<W^ | **<0.0001** | 0.17 | MCTQ_SD_ | -0.14 | (-0.83, 0.55) | 0.695 | 0.754 | 0.00 | 0.15 (0.16) |
| MSF_corr_ | 0.03 | (-0.62, 0.68) | 0.935 | 0.610 | 0.00 | PSQI_g_ | -2.19 | (-2.59, -1.79) | **<0.0001**^M<W^ | **<0.0001** | 0.19 | BSS_SD_ | -0.85 | (-1.74, 0.04) | 0.062 | 0.075 | 0.01 | 0.16 (0.18) |
| MSF_corr_ | 0.21 | (-0.44, 0.86) | 0.526 | 0.981 | 0.00 | PSQI_g_ | -2.50 | (-2.93, -2.08) | **<0.0001**^M<W^ | **<0.0001** | 0.22 | PSQI_SD_ | -1.69 | (-2.51, -0.87) | **<0.0001** | **<0.0001** | 0.03 | 0.18 (0.20) |

Regression models including the combinations of multiple outcome measures of chronotype, sleep quality and sleep duration as predictors and general mental health as dependent variable. Second p value indicates significance after controlling for additional covariates: sex, age, ethnicity, work status, gross income, marital status and alcohol consumption. P values in bold indicate significant effects (p < 0.05). Superscript letters indicate if p-values were significant when data for ^M^men and ^W^women were analysed separately, with </>/≃ signs to indicate which sex group had a greater/similar effect size. MEQ- Morningness-Evenigness Questionnaire total score, MCTQ_Iam_- Munich Chronotype Questionnaire diurnal preference, MSF_corr_- Midpoint of sleep on free days corrected for corrected for the sleep debt accumulated during the week (see methods), PSQI_sq_- Pittsburgh Sleep Quality Index single question, PSQI_g_- Pittsburgh Sleep Quality Index global score, ISI- Insomnia Severity Index total score, MCTQ_SD_- Munich Chronotype Questionnaire based on work and free days, BSS_SD_- British Sleep Survey sleep duration based on last week, PSQI_SD_- Pittsburgh Sleep Quality Index sleep duration based on the last month. According to Cohen’s (1988) guidelines *f^2^*≥ 0.02, *f^2^*≥ 0.15, and *f^2^*≥ 0.35 represent small, medium, and large effect sizes, respectively.

**Supplementary Table 3c:** The independent contribution of Chronotype, Sleep quality and Sleep duration on **physical health** across all models of different predictors combinations

| **Chronotype** | | | | | | **Sleep quality** | | | | | | **Sleep duration** | | | | | | Model R^2^ without (with  covariates) |
| --- | --- | --- | --- | --- | --- | --- | --- | --- | --- | --- | --- | --- | --- | --- | --- | --- | --- | --- |
| Used measure | β | 95% CI | p value | p-value  controlled | *f^2^* | Used measure | β | 95% CI | p-value | p-value  controlled | *f^2^* | Used  measure | β | 95% CI | p-value | p-value  controlled | *f^2^* |  |
| MEQ | 0.04 | (-0.03, 0.11) | 0.233 | 0.443 | 0.00 | PSQI_sq_ | -3.17 | (-4.22, -2.12) | **<0.0001**^M<W^ | **<0.0001** | 0.06 | MCTQ_SD_ | -0.11 | (-0.66, 0.45) | 0.710 | 0.717 | 0.00 | 0.05 (0.07) |
| MEQ | 0.06 | (-0.01, 0.13) | 0.095 | 0.305 | 0.00 | PSQI_sq_ | -3.59 | (-4.66, -2.52) | **<0.0001**^M<W^ | **<0.0001** | 0.07 | BSS_SD_ | -0.40 | (-1.11, 0.32) | 0.273 | 0.740 | 0.00 | 0.07 (0.08) |
| MEQ | 0.05 | (-0.02, 0.12) | 0.166 | 0.389 | 0.00 | PSQI_sq_ | -3.34 | (-4.40, -2.28) | **<0.0001**^M<W^ | **<0.0001** | 0.06 | PSQI_SD_ | -0.28 | (-0.89, 0.33) | 0.365 | 0.842 | 0.00 | 0.06 (0.07) |
| MEQ | -0.02 | (-0.09, 0.05) | 0.607 | 0.017 | 0.00 | ISI | -0.93 | (-1.12, -0.75) | **<0.0001**^M<W^ | **<0.0001** | 0.16 | MCTQ_SD_ | -0.10 | (-0.64, 0.44) | 0.726 | 0.751 | 0.00 | 0.14 (0.14) |
| MEQ | -0.01 | (-0.08, 0.07) | 0.884 | 0.457 | 0.00 | ISI | -0.96 | (-1.14, -0.78) | **<0.0001**^M<W^ | **<0.0001** | 0.17 | BSS_SD_ | -0.31 | (-1.00, 0.37) | 0.368 | 0.920 | 0.00 | 0.15 (0.15) |
| MEQ | -0.02 | (-0.09, 0.06) | 0.665 | 0.368 | 0.00 | ISI | -0.98 | (-1.16, -0.80) | **<0.0001**^M<W^ | **<0.0001** | 0.17 | PSQI_SD_ | -0.27 | (-0.85, 0.31) | 0.358 | 0.950 | 0.00 | 0.15 (0.15) |
| MEQ | 0.00 | (-0.07, 0.08) | 0.951 | 0.983 | 0.00 | PSQI_g_ | -1.28 | (-1.61, -0.96) | **<0.0001**^M<W^ | **<0.0001** | 0.10 | MCTQ_SD_ | -0.08 | (-0.64, 0.49) | 0.782 | 0.915 | 0.00 | 0.09 (0.10) |
| MEQ | 0.01 | (-0.06, 0.09) | 0.762 | 0.869 | 0.00 | PSQI_g_ | -1.39 | (-1.73, -1.06) | **<0.0001**^M<W^ | **<0.0001** | 0.11 | BSS_SD_ | -0.59 | (-1.34, 0.16) | 0.122^M^ | 0.164 | 0.00 | 0.10 (0.11) |
| MEQ | -0.01 | (-0.08, 0.07) | 0.812 | 0.811 | 0.00 | PSQI_g_ | -1.59 | (-1.94, -1.23) | **<0.0001**^M<W^ | **<0.0001** | 0.13 | PSQI_SD_ | -1.16 | (-1.85, -0.48) | **<0.0001**^W^ | **0.004** | 0.02 | 0.11 (0.12) |
| MCTQ_Iam_ | 0.01 | (-0.36, 0.38) | 0.955 | 0.851 | 0.00 | PSQI_sq_ | -3.32 | (-4.36, -2.28) | **<0.0001**^M<W^ | **<0.0001** | 0.06 | MCTQ_SD_ | -0.12 | (-0.68, 0.44) | 0.665 | 0.814 | 0.00 | 0.05 (0.07) |
| MCTQ_Iam_ | -0.09 | (-0.46, 0.28) | 0.630 | 0.938 | 0.00 | PSQI_sq_ | -3.73 | (-4.79, -2.66) | **<0.0001**^M<W^ | **<0.0001** | 0.07 | BSS_SD_ | -0.38 | (-1.09, 0.32) | 0.289 | 0.813 | 0.00 | 0.07 (0.08) |
| MCTQ_Iam_ | -0.03 | (-0.40, 0.34) | 0.869 | 0.932 | 0.00 | PSQI_sq_ | -3.48 | (-4.54, -2.43) | **<0.0001**^M<W^ | **<0.0001** | 0.06 | PSQI_SD_ | -0.32 | (-0.92, 0.29) | 0.309 | 0.885 | 0.00 | 0.06 (0.07) |
| MCTQ_Iam_ | 0.20 | (-0.16, 0.57) | 0.270^M^ | 0.206 | 0.00 | ISI | -0.93 | (-1.11, -0.75) | **<0.0001**^M<W^ | **<0.0001** | 0.17 | MCTQ_SD_ | -0.10 | (-0.65, 0.44) | 0.706 | 0.934 | 0.00 | 0.14 (0.14) |
| MCTQ_Iam_ | 0.13 | (-0.23, 0.49) | 0.483 | 0.260 | 0.00 | ISI | -0.96 | (-1.14, -0.78) | **<0.0001**^M<W^ | **<0.0001** | 0.18 | BSS_SD_ | -0.23 | (-0.91, 0.45) | 0.512 | 0.904 | 0.00 | 0.15 (0.15) |
| MCTQ_Iam_ | 0.18 | (-0.18, 0.54) | 0.325^M^ | 0.198 | 0.00 | ISI | -0.98 | (-1.15, -0.80) | **<0.0001**^M<W^ | **<0.0001** | 0.18 | PSQI_SD_ | -0.27 | (-0.85, 0.32) | 0.372 | 0.930 | 0.00 | 0.15 (0.15) |
| MCTQ_Iam_ | 0.20 | (-0.18, 0.58) | 0.291^M^ | 0.294 | 0.00 | PSQI_g_ | -1.32 | (-1.64, -1.00) | **<0.0001**^M<W^ | **<0.0001** | 0.11 | MCTQ_SD_ | -0.12 | (-0.69, 0.44) | 0.672 | 0.934 | 0.00 | 0.09 (0.10) |
| MCTQ_Iam_ | 0.13 | (-0.25, 0.51) | 0.489 | 0.404 | 0.00 | PSQI_g_ | -1.43 | (-1.76, -1.09) | **<0.0001**^M<W^ | **<0.0001** | 0.12 | BSS_SD_ | -0.58 | (-1.32, 0.17) | 0.128^M^ | 0.199 | 0.00 | 0.10 (0.11) |
| MCTQ_Iam_ | 0.25 | (-0.13, 0.63) | 0.199^M^ | 0.207 | 0.00 | PSQI_g_ | -1.63 | (-1.98, -1.27) | **<0.0001**^M<W^ | **<0.0001** | 0.13 | PSQI_SD_ | -1.23 | (-1.90, -0.55) | **0.0004**^M>W^ | **0.003** | 0.02 | 0.11 (0.12) |
| MSF_corr_ | 0.24 | (-0.28, 0.75) | 0.367 | 0.184 | 0.00 | PSQI_sq_ | -3.38 | (-4.43, -2.34) | **<0.0001**^M<W^ | **<0.0001** | 0.06 | MCTQ_SD_ | -0.18 | (-0.74, 0.38) | 0.534 | 0.927 | 0.00 | 0.05 (0.07) |
| MSF_corr_ | 0.09 | (-0.43, 0.61) | 0.729 | 0.312 | 0.00 | PSQI_sq_ | -3.77 | (-4.84, -2.71) | **<0.0001**^M<W^ | **<0.0001** | 0.07 | BSS_SD_ | -0.38 | (-1.09, 0.32) | 0.287 | 0.815 | 0.00 | 0.07 (0.08) |
| MSF_corr_ | 0.15 | (-0.37, 0.66) | 0.576 | 0.260 | 0.00 | PSQI_sq_ | -3.53 | (-4.59, -2.47) | **<0.0001**^M<W^ | **<0.0001** | 0.06 | PSQI_SD_ | -0.34 | (-0.95, 0.27) | 0.274 | 0.913 | 0.00 | 0.06 (0.07) |
| MSF_corr_ | 0.47 | (-0.03, 0.98) | 0.066^M^ | **0.041** | 0.01 | ISI | -0.95 | (-1.13, -0.77) | **<0.0001**^M<W^ | **<0.0001** | 0.17 | MCTQ_SD_ | -0.15 | (-0.70, 0.39) | 0.587 | 0.982 | 0.00 | 0.14 (0.14) |
| MSF_corr_ | 0.38 | (-0.13, 0.89) | 0.144^M^ | 0.054 | 0.00 | ISI | -0.97 | (-1.15, -0.80) | **<0.0001**^M<W^ | **<0.0001** | 0.18 | BSS_SD_ | -0.25 | (-0.93, 0.43) | 0.476 | 0.912 | 0.00 | 0.15 (0.15) |
| MSF_corr_ | 0.44 | (-0.06, 0.94) | 0.087^M^ | **0.040** | 0.00 | ISI | -0.99 | (-1.17, -0.81) | **<0.0001**^M<W^ | **<0.0001** | 0.18 | PSQI_SD_ | -0.30 | (-0.88, 0.28) | 0.314 | 0.974 | 0.00 | 0.15 (0.15) |
| MSF_corr_ | 0.56 | (0.03, 1.10) | **0.040**^M^ | **0.034** | 0.01 | PSQI_g_ | -1.36 | (-1.69, -1.04) | **<0.0001**^M<W^ | **<0.0001** | 0.12 | MCTQ_SD_ | -0.19 | (-0.76, 0.37) | 0.500 | 0.795 | 0.00 | 0.10 (0.11) |
| MSF_corr_ | 0.46 | (-0.09, 1.00) | 0.099^M^ | 0.062 | 0.00 | PSQI_g_ | -1.47 | (-1.80, -1.13) | **<0.0001**^M<W^ | **<0.0001** | 0.12 | BSS_SD_ | -0.64 | (-1.38, 0.11) | 0.095^M^ | 0.169 | 0.00 | 0.11 (0.12) |
| MSF_corr_ | 0.68 | (0.14, 1.22) | **0.014**^M^ | **0.015** | 0.01 | PSQI_g_ | -1.70 | (-2.06, -1.34) | **<0.0001**^M<W^ | **<0.0001** | 0.14 | PSQI_SD_ | -1.34 | (-2.02, -0.66) | **0.0001**^M<W^ | **0.002** | 0.02 | 0.12 (0.13) |

Regression models including the combinations of multiple outcome measures of chronotype, sleep quality and sleep duration as predictors and physical health as dependent variable. Second p value indicates significance after controlling for additional covariates: sex, age, ethnicity, work status, gross income, marital status and alcohol consumption. P values in bold indicate significant effects (p < 0.05). Superscript letters indicate if p-values were significant when data for ^M^men and ^W^women were analysed separately, with </>/≃ signs to indicate which sex group had a greater/similar effect size. MEQ- Morningness-Evenigness Questionnaire total score, MCTQ_Iam_- Munich Chronotype Questionnaire diurnal preference, MSF_corr_- Midpoint of sleep on free days corrected for corrected for the sleep debt accumulated during the week (see methods), PSQI_sq_- Pittsburgh Sleep Quality Index single question, PSQI_g_- Pittsburgh Sleep Quality Index global score, ISI- Insomnia Severity Index total score, MCTQ_SD_- Munich Chronotype Questionnaire based on work and free days, BSS_SD_- British Sleep Survey sleep duration based on last week, PSQI_SD_- Pittsburgh Sleep Quality Index sleep duration based on the last month. According to Cohen’s (1988) guidelines *f^2^*≥ 0.02, *f^2^*≥ 0.15, and *f^2^*≥ 0.35 represent small, medium, and large effect sizes, respectively.

**Supplementary Table 4a:** The Independent Contribution of Chronotype, Sleep Quality (ISI) and Sleep Duration on Health in Men and Women

| Physical and Psychological Wellbeing Variables | Chronotype | | | | |  | Sleep Quality | | | | Sleep Duration | | | | |  |
| --- | --- | --- | --- | --- | --- | --- | --- | --- | --- | --- | --- | --- | --- | --- | --- | --- |
|  | Beta | 95.0 Confidence Interval for B | | p- value | Cohens  *f^2^* | Beta | 95.0 Confidence Interval for B | | p- value | Cohens *f^2^* | Beta | 95.0 Confidence Interval for B | | p-value | Cohens *f^2^* | Adjusted  R Square |
| **Men** | | | | | | | | | | | | | | | | |
| Psychiatric Health | -0.071 | -0.115 | -0.027 | **0.002** | 0.030 | 0.313 | 0.199 | 0.427 | **<0.0001** | 0.086 | 0.192 | -0.129 | 0.513 | 0.241 | 0.004 | 0.142 |
| Mental Health | 0.073 | -0.041 | 0.187 | 0.208 | 0.005 | -1.112 | -1.405 | -0.818 | **<0.0001** | 0.161 | 0.005 | -0.821 | 0.067 | 0.854 | 0.000 | 0.150 |
| Physical Health | -0.065 | -0.161 | 0.031 | 0.185 | 0.005 | -0.911 | -1.159 | -0.664 | **<0.0001** | 0.152 | -0.144 | -0.840 | 0.553 | 0.685 | 0.000 | 0.135 |
| **Women** | | | | | | | | | | | | | | | | |
| Psychiatric Health | -0.022 | -0.083 | 0.040 | 0.490 | 0.002 | 0.433 | 0.287 | 0.579 | **<0.0001** | 0.154 | -0.053 | -0.508 | 0.402 | 0.817 | 0.000 | 0.165 |
| Mental Health | 0.183 | 0.030 | 0.337 | **0.019** | 0.025 | -1.488 | -1.856 | -1.119 | **<0.0001** | 0.280 | 0.170 | -0.978 | 0.096 | 0.934 | 0.000 | 0.259 |
| Physical Health | -0.002 | -0.134 | 0.130 | 0.980 | 0.000 | -0.926 | -1.243 | -0.609 | **<0.0001** | 0.146 | 0.304 | -0.685 | 1.293 | 0.546 | 0.002 | 0.127 |

Regression model including chronotype, sleep quality and sleep duration as predictors and measures of physical and mental health and psychological wellbeing as dependent variables. Chronotype is measured as the total score of the Morningness-Evenigness Questionnaire (MEQ) where higher score indicates greater morning preference. Sleep quality is measured as the total score from the Insomnia Severity Index (ISI), where a higher score indicates poorer sleep quality. Sleep duration is measured as the average sleep period time across work days and free days from the Munich Chronotype Questionnaire (MCTQ) where an increasing score indicates a longer sleep duration. Higher scores of general psychiatric health indicate poorer health, whilst lower scores in physical and mental health indicate poorer health outcomes. According to Cohen’s (1988) guidelines *f^2^*≥ 0.02, *f^2^*≥ 0.15, and *f^2^*≥ 0.35 represent small, medium, and large effect sizes, respectively. Analyses are controlled for additional covariates: Sex, Age, ethnicity, work status, gross income, marital status and alcohol consumption. . P values in bold indicate significant effects (p < 0.05).

**Supplementary Table 4b:** The Independent Contribution of Chronotype, Sleep Quality (PSQIglobal) and Sleep Duration on Health in Men and Women.

| Physical and Psychological Wellbeing Variables | Chronotype | | | | |  | Sleep Quality | | | | Sleep Duration | | | | |  |
| --- | --- | --- | --- | --- | --- | --- | --- | --- | --- | --- | --- | --- | --- | --- | --- | --- |
|  | Beta | 95.0 Confidence Interval for B | | p- value | Cohens  *f^2^* | Beta | 95.0 Confidence Interval for B | | p- value | Cohens *f^2^* | Beta | 95.0 Confidence Interval for B | | p-value | Cohens *f^2^* | Adjusted  R Square |
| **Men** | | | | | | | | | | | | | | | | |
| Psychiatric Health | -0.097 | -0.141 | -0.053 | **<0.0001** | 0.057 | 0.338 | 0.153 | 0.523 | **<0.0001** | 0.040 | 0.214 | -0.124 | 0.552 | 0.215 | 0.005 | 0.112 |
| Mental Health | 0.155 | 0.041 | 0.269 | **0.008** | 0.022 | -1.497 | -1.973 | -1.020 | **<0.0001** | 0.116 | -0.046 | -0.915 | 0.054 | 0.541 | 0.001 | 0.119 |
| Physical Health | -0.008 | -0.103 | 0.087 | 0.872 | 0.000 | -1.183 | -1.580 | -0.786 | **<0.0001** | 0.105 | -0.062 | -0.786 | 0.662 | 0.867 | 0.000 | 0.090 |
| **Women** | | | | | | | | | | | | | | | | |
| Psychiatric Health | -0.042 | -0.107 | 0.024 | 0.215 | 0.007 | 0.581 | 0.291 | 0.870 | **<0.0001** | 0.071 | 0.013 | -0.471 | 0.496 | 0.959 | 0.000 | 0.098 |
| Mental Health | 0.225 | 0.065 | 0.384 | **0.006** | 0.034 | -2.653 | -3.363 | -1.944 | **<0.0001** | 0.243 | -0.426 | -1.608 | 0.093 | 0.795 | 0.000 | 0.230 |
| Physical Health | 0.000 | -0.139 | 0.138 | 0.996 | 0.000 | -1.558 | -2.175 | -0.942 | **<0.0001** | 0.111 | 0.063 | -0.965 | 1.091 | 0.904 | 0.000 | 0.097 |

Regression model including chronotype, sleep quality and sleep duration as predictors and measures of physical and mental health and psychological wellbeing as dependent variables. Chronotype is measured as the total score of the Morningness-Evenigness Questionnaire (MEQ) where higher score indicates greater morning preference. Sleep quality is measured as the global score from the Pittsburgh Sleep Quality Index (PSQI), where a higher score indicates poorer sleep quality. Sleep duration is measured as the average sleep period time across work days and free days from the Munich Chronotype Questionnaire (MCTQ) where an increasing score indicates a longer sleep duration. Higher scores of general psychiatric health indicate poorer health, whilst lower scores in physical and mental health indicate poorer health outcomes. According to Cohen’s (1988) guidelines *f^2^*≥ 0.02, *f^2^*≥ 0.15, and *f^2^*≥ 0.35 represent small, medium, and large effect sizes, respectively. Analyses are controlled for additional covariates: Sex, Age, ethnicity, work status, gross income, marital status and alcohol consumption. P values in bold indicate significant effects (p < 0.05).

**Supplementary Table 4c:** The Independent Contribution of Chronotype, Sleep Quality (PSQI single question) and Sleep Duration on Health in Men and Women

| Physical and Psychological Wellbeing Variables | Chronotype | | | | |  | Sleep Quality | | | | Sleep Duration | | | | |  |
| --- | --- | --- | --- | --- | --- | --- | --- | --- | --- | --- | --- | --- | --- | --- | --- | --- |
|  | Beta | 95.0 Confidence Interval for B | | p- value | Cohens  *f^2^* | Beta | 95.0 Confidence Interval for B | | p- value | Cohens *f^2^* | Beta | 95.0 Confidence Interval for B | | p-value | Cohens *f^2^* | Adjusted  R Square |
| **Men** | | | | | | | | | | | | | | | | |
| Psychiatric Health | -0.097 | -0.141 | -0.053 | **<0.0001** | 0.057 | 0.338 | 0.153 | 0.523 | **<0.0001** | 0.040 | 0.214 | -0.124 | 0.552 | 0.215 | 0.005 | 0.112 |
| Mental Health | 0.155 | 0.041 | 0.269 | **0.008** | 0.022 | -1.497 | -1.973 | -1.020 | **<0.0001** | 0.116 | -0.046 | -0.915 | 0.054 | 0.541 | 0.001 | 0.119 |
| Physical Health | -0.008 | -0.103 | 0.087 | 0.872 | 0.000 | -1.183 | -1.580 | -0.786 | **<0.0001** | 0.105 | -0.062 | -0.786 | 0.662 | 0.867 | 0.000 | 0.090 |
| **Women** | | | | | | | | | | | | | | | | |
| Psychiatric Health | -0.053 | -0.115 | 0.009 | 0.094 | 0.012 | 1.820 | 0.968 | 2.672 | **<0.0001** | 0.077 | -0.114 | -0.581 | 0.353 | 0.630 | 0.001 | 0.105 |
| Mental Health | 0.278 | 0.119 | 0.437 | **0.001** | 0.050 | -6.143 | -8.338 | -3.948 | **<0.0001** | 0.129 | 0.207 | -0.999 | 0.097 | 0.908 | 0.000 | 0.155 |
| Physical Health | 0.028 | -0.101 | 0.158 | 0.666 | 0.001 | -4.831 | -6.621 | -3.040 | **<0.0001** | 0.120 | 0.357 | -0.627 | 1.341 | 0.476 | 0.002 | 0.107 |

Regression model including chronotype, sleep quality and sleep duration as predictors and measures of physical and mental health and psychological wellbeing as dependent variables. Chronotype is measured as the total score of the Morningness-Evenigness Questionnaire (MEQ) where higher score indicates greater morning preference. Sleep quality is measured as the single sleep quality question from the Pittsburgh Sleep Quality Index (PSQI), where a higher score indicates poorer sleep quality. Sleep duration is measured as the average sleep period time across work days and free days from the Munich Chronotype Questionnaire (MCTQ) where an increasing score indicates a longer sleep duration. Higher scores of general psychiatric health indicate poorer health, whilst lower scores in physical and mental health indicate poorer health outcomes. According to Cohen’s (1988) guidelines *f^2^*≥ 0.02, *f^2^*≥ 0.15, and *f^2^*≥ 0.35 represent small, medium, and large effect sizes, respectively. Analyses are controlled for additional covariates: Sex, Age, ethnicity, work status, gross income, marital status and alcohol consumption. P values in bold indicate significant effects (p < 0.05).

**Supplementary Table 4d:** The Independent Contribution of Chronotype, Sleep Quality (KSD) and Sleep Duration on Health in Men and Women

| Physical and Psychological Wellbeing Variables | Chronotype | | | | |  | Sleep Quality | | | | Sleep Duration | | | | |  |
| --- | --- | --- | --- | --- | --- | --- | --- | --- | --- | --- | --- | --- | --- | --- | --- | --- |
|  | Beta | 95.0 Confidence Interval for B | | p- value | Cohens  *f^2^* | Beta | 95.0 Confidence Interval for B | | p- value | Cohens *f^2^* | Beta | 95.0 Confidence Interval for B | | p-value | Cohens *f^2^* | Adjusted  R Square |
| **Men** | | | | | | | | | | | | | | | | |
| Psychiatric Health | -0.090 | -0.133 | -0.048 | **<0.0001** | 0.050 | 0.515 | 0.286 | 0.744 | **<0.0001** | 0.057 | 0.154 | -0.166 | 0.475 | 0.344 | 0.003 | 0.122 |
| Mental Health | 0.157 | 0.044 | 0.271 | **0.007** | 0.021 | -1.562 | -2.172 | -0.953 | **<0.0001** | 0.073 | 0.162 | -0.692 | 0.044 | 0.433 | 0.002 | 0.083 |
| Physical Health | 0.022 | -0.075 | 0.120 | 0.652 | 0.001 | -0.655 | -1.181 | -0.129 | **0.015** | 0.017 | -0.006 | -0.743 | 0.730 | 0.987 | 0.000 | 0.020 |
| **Women** | | | | | | | | | | | | | | | | |
| Psychiatric Health | -0.051 | -0.113 | 0.012 | 0.110 | 0.011 | 0.764 | 0.391 | 1.136 | **<0.0001** | 0.072 | -0.125 | -0.598 | 0.348 | 0.603 | 0.001 | 0.099 |
| Mental Health | 0.273 | 0.113 | 0.434 | **0.001** | 0.049 | -2.617 | -3.581 | -1.653 | **<0.0001** | 0.124 | 0.228 | -0.997 | 0.113 | 0.897 | 0.000 | 0.144 |
| Physical Health | 0.059 | -0.070 | 0.188 | 0.370 | 0.004 | -1.892 | -2.667 | -1.117 | **<0.0001** | 0.101 | 0.336 | -0.648 | 1.321 | 0.502 | 0.002 | 0.096 |

Regression model including chronotype, sleep quality and sleep duration as predictors and measures of physical and mental health and psychological wellbeing as dependent variables. Chronotype is measured as the total score of the Morningness-Evenigness Questionnaire (MEQ) where higher score indicates greater morning preference. Sleep quality is measured from the Karolinska Sleep Diary (KSD) single sleep quality question, where a higher score indicates poorer sleep quality. Sleep duration is measured as the average sleep period time across work days and free days from the Munich Chronotype Questionnaire (MCTQ) where an increasing score indicates a longer sleep duration. Higher scores of general psychiatric health indicate poorer health, whilst lower scores in physical and mental health indicate poorer health outcomes. According to Cohen’s (1988) guidelines *f^2^*≥ 0.02, *f^2^*≥ 0.15, and *f^2^*≥ 0.35 represent small, medium, and large effect sizes, respectively. Analyses are controlled for additional covariates: Sex, Age, ethnicity, work status, gross income, marital status and alcohol consumption. P values in bold indicate significant effects (p < 0.05).

**Supplementary Table 5:** Sex differences in the Association between Chronotype, Sleep Quality and Duration with Various Health Measures.

| **Health Variable** | **Pearson Correlation Data** | | | | | | | | **Fisher Transformation** | | |
| --- | --- | --- | --- | --- | --- | --- | --- | --- | --- | --- | --- |
|  | **Men** | | | | **Women** | | | | z | p | q |
|  | N | R | p | q | N | R | p | q |  |  |  |
| **Psychiatric Health**  Chronotype  Sleep Quality  Sleep Duration | 401  399  392 | -0.252  0.338  0.083 | **<0.0001**  **<0.0001**  0.103 | **<0.0001**  **<0.0001**  0.185 | 258  249  257 | -0.142  0.374  -0.066 | 0.024  **<0.0001**  0.299 | 0.064  **<0.0001**  0.418 | -1.43  -0.51  1.85 | 0.076  0.305  0.032 | 0.160  0.419  0.079 |
| **Physical Health**  Chronotype  Sleep Quality  Sleep Duration | 401  399  392 | 0.084  -0.351  -0.025 | 0.093  **<0.0001**  0.627 | 0.171  **<0.0001**  0.698 | 258  249  257 | 0.120  -0.435  0.039 | 0.055  **<0.0001**  0.529 | 0.124  **<0.0001**  0.605 | -0.45  1.23  0.17 | 0.326  0.109  0.433 | 0.435  0.181  0.521 |
| **Mental Health**  Chronotype  Sleep Quality  Sleep Duration | 401  399  392 | 0.161  -0.407  0.006 | **0.001**  **<0.0001**  0.907 | 0.004  **<0.0001**  0.939 | 258  249  257 | 0.263  -0.541  0.050 | **<0.0001**  **<0.0001**  0.426 | **<0.0001**  **<0.0001**  0.521 | -1.33  2.14  -0.55 | 0.092  **0.016**  0.291 | 0.172  **0.045**  0.420 |

Pearson correlation analysis of chronotype, sleep quality and sleep duration for each gender and Fisher transformation analysis of correlation figures. Chronotype measure is based on TotalHO scores of the Morningness-Evenigness Questionnaire (MEQ), Sleep quality measure is from the Insomnia Severity Index (ISI) and Sleep duration measure is based on the average time spent in bed calculation from the Munich Chronotype Questionnaire (MCTQ). General psychiatric health measure is from General Health Questionnaire (GHQ). Physical and mental health measures are calculated from the Short From-36 Health Questionnaire (SF36).

**Supplementary Table 6:** The Independent Contribution of Chronotype, Sleep Quality, Sleep Duration and Social Jetlag on Measures of Health and Psychological Characteristics

| Physical and Psychological Wellbeing Variables | Chronotype (MEQ) | | | | | Sleep Quality (PSQI_sq_) | | | | | | | | Sleep Duration (MCTQ_SD_) | | | | | Social JetLag | | | | |
| --- | --- | --- | --- | --- | --- | --- | --- | --- | --- | --- | --- | --- | --- | --- | --- | --- | --- | --- | --- | --- | --- | --- | --- |
|  | Beta | 95% Confidence Interval | P-value | *f^2^* | Beta | | | 95% Confidence Interval | | P-value | | *f^2^* | Beta | | 95% Confidence Interval | P-value | *f^2^* | Beta | | 95% Confidence Interval | P-value | *f^2^* |  |
| Psychiatric Health | -0.09 | (-0.12, -0.06) | **<.0001** | 0.042 | 1.50 | | (1.02, 1.99) | | **<.0001** | | 0.059 | | 0.08 | | (-0.18, 0.34) | 0.543 | 0.001 | -0.39 | | (-0.71, -0.08) | **0.013** | 0.010 |  |
| Mental Health | 0.20 | (0.11, 0.29) | **<.0001** | 0.031 | -4.47 | | (-5.76, -3.18) | | **<.0001** | | 0.073 | | 0.09 | | (-0.59, 0.78) | 0.790 | 0.000 | 0.13 | | (-0.70, 0.95) | 0.766 | 0.000 |  |
| Physical Health | 0.04 | (-0.03, 0.12) | 0.257 | 0.002 | -3.16 | | (-4.21, -2.11) | | **<.0001** | | 0.055 | | -0.09 | | (-0.65, 0.46) | 0.743 | 0.000 | 0.00 | | (-0.67, 0.68) | 0.991 | 0.000 |  |
| BMI | 0.01 | (-0.02, 0.04) | 0.685 | 0.000 | 0.00 | | (-0.42, 0.43) | | 0.999 | | 0.000 | | -0.18 | | (-0.40, 0.05) | 0.119 | 0.004 | 0.04 | | (-0.23, 0.31) | 0.780 | 0.000 |  |
| ***DEBQ*** |  |  |  |  |  | |  | |  | |  | |  | |  |  |  |  | |  |  |  |  |
| Restrained Eating | 0.01 | (0.00, 0.02) | **0.026** | 0.008 | 0.06 | | (-0.06, 0.18) | | 0.312 | | 0.002 | | 0.02 | | (-0.04, 0.08) | 0.467 | 0.001 | 0.06 | | (-0.01, 0.14) | 0.097 | 0.004 |  |
| Emotional Eating | -0.01 | (-0.02, 0.00) | **0.034** | 0.007 | 0.10 | | (0.00, 0.21) | | 0.061 | | 0.006 | | -0.01 | | (-0.06, 0.05) | 0.835 | 0.000 | 0.06 | | (0.00, 0.13) | 0.059 | 0.006 |  |
| External Eating | -0.02 | (-0.02, -0.01) | **<.0001** | 0.040 | 0.03 | | (-0.06, 0.13) | | 0.467 | | 0.001 | | -0.01 | | (-0.06, 0.04) | 0.599 | 0.000 | 0.07 | | (0.01, 0.13) | **0.027** | 0.008 |  |
| ***Big Five Inventory*** |  |  |  |  |  | |  | |  | |  | |  | |  |  |  |  | |  |  |  |  |
| Openness | -0.04 | (-0.09, 0.02) | 0.185 | 0.003 | -0.83 | | (-1.59, -0.07) | | **0.033** | | 0.007 | | -0.19 | | (-0.59, 0.22) | 0.362 | 0.001 | 0.04 | | (-0.45, 0.53) | 0.874 | 0.000 |  |
| Conscientiousness | 0.24 | (0.19, 0.29) | **<.0001** | 0.130 | -0.43 | | (-1.19, 0.32) | | 0.262 | | 0.002 | | 0.22 | | (-0.18, 0.63) | 0.275 | 0.002 | 0.20 | | (-0.28, 0.68) | 0.418 | 0.001 |  |
| Extraversion | 0.05 | (0.00, 0.10) | 0.056 | 0.006 | -0.95 | | (-1.68, -0.22) | | **0.011** | | 0.010 | | 0.10 | | (-0.29, 0.49) | 0.603 | 0.000 | 0.03 | | (-0.44, 0.50) | 0.904 | 0.000 |  |
| Agreeableness | 0.12 | (0.07, 0.17) | **<.0001** | 0.039 | -1.48 | | (-2.16, -0.79) | | **<.0001** | | 0.029 | | 0.46 | | (0.10, 0.83) | **0.012** | 0.010 | 0.37 | | (-0.06, 0.81) | 0.094 | 0.004 |  |
| Neuroticism | -0.10 | (-0.15, -0.05) | **0.000** | 0.023 | 2.45 | | (1.70, 3.20) | | **<.0001** | | 0.066 | | 0.09 | | (-0.31, 0.49) | 0.652 | 0.000 | -0.05 | | (-0.53, 0.43) | 0.838 | 0.000 |  |
| ***PANAS*** |  |  |  |  |  | |  | |  | |  | |  | |  |  |  |  | |  |  |  |  |
| Positive Affect | 0.17 | (0.11, 0.23) | **<.0001** | 0.049 | -1.37 | | (-2.24, -0.50) | | **0.002** | | 0.015 | | -0.30 | | (-0.77, 0.16) | 0.203 | 0.003 | 0.23 | | (-0.33, 0.79) | 0.413 | 0.001 |  |
| Negative Affect | -0.07 | (-0.12, -0.02) | **0.011** | 0.011 | 1.86 | | (1.14, 2.57) | | **<.0001** | | 0.042 | | -0.11 | | (-0.49, 0.28) | 0.586 | 0.000 | 0.04 | | (-0.42, 0.50) | 0.872 | 0.000 |  |
| ***BIS-BAS*** |  |  |  |  |  | |  | |  | |  | |  | |  |  |  |  | |  |  |  |  |
| BIS | 0.02 | (-0.02, 0.06) | 0.257 | 0.002 | -0.76 | | (-1.28, -0.24) | | **0.004** | | 0.013 | | -0.05 | | (-0.32, 0.23) | 0.735 | 0.000 | -0.25 | | (-0.59, 0.08) | 0.136 | 0.004 |  |
| BAS reward | -0.02 | (-0.04, 0.00) | **0.029** | 0.008 | 0.13 | | (-0.15, 0.41) | | 0.369 | | 0.001 | | 0.03 | | (-0.12, 0.18) | 0.686 | 0.000 | -0.15 | | (-0.33, 0.03) | 0.110 | 0.004 |  |
| BAS drive | -0.03 | (-0.05, 0.00) | **0.023** | 0.008 | 0.33 | | (0.02, 0.65) | | **0.038** | | 0.007 | | 0.05 | | (-0.12, 0.21) | 0.571 | 0.001 | -0.16 | | (-0.37, 0.04) | 0.109 | 0.004 |  |
| BAS fun seeking | 0.02 | (0.00, 0.04) | 0.080 | 0.005 | 0.32 | | (0.03, 0.62) | | **0.033** | | 0.007 | | 0.01 | | (-0.15, 0.17) | 0.893 | 0.000 | -0.19 | | (-0.38, 0.00) | **0.049** | 0.006 |  |

Regression model including chronotype, sleep quality, sleep duration and social jetlag as predictors and measures of physical and mental health and psychological wellbeing as dependent variables. Chronotype is measured as the total score of the Morningness-Evenigness Questionnaire (MEQ) where higher score indicates greater morning preference. Sleep quality is measured as the single sleep quality question from the Pittsburgh Sleep Quality Index (PSQI_sq_), where a higher score indicates poorer sleep quality. Sleep duration is measured as the average sleep period time across work days and free days from the Munich Chronotype Questionnaire (MCTQ_SD_) where an increasing score indicates a longer sleep duration. Social Jet lag is measured as the absolute difference between the midpoint of sleep during free-days and workdays as derived from the MCTQ. Higher scores of general psychiatric health indicate poorer health, whilst lower scores in physical and mental health indicate poorer health outcomes. Higher depression scores indicate greater risk of depression. For the Dutch Eating Behaviour Questionnaire (DEBQ ) higher scores indicate greater endorsement of the eating behaviour, for the Big Five Inventory a higher score indicates the stronger presence of a personality trait, the Positive and negative affect scale (PANAS) higher scores indicate greater positive or negative engagement with the environment, and for the Behavioural Inhibition and Approach systems questionnaire (BIS-BAS) lower scores indicate a greater drive through the anticipation of either punishment or reward. According to Cohen’s (1988) guidelines *f^2^*≥ 0.02, *f^2^*≥ 0.15, and *f^2^*≥ 0.35 represent small, medium, and large effect sizes, respectively. Analyses are controlled for additional covariates: Sex, Age, ethnicity, work status, gross income, marital status and alcohol consumption. P values in bold indicate significant effects (p < 0.05).

**Supplementary Figure 1:** The Gender Differences in Local Effect of Chronotype, Sleep Duration and four different Sleep Quality measures on Health Measures

**Men
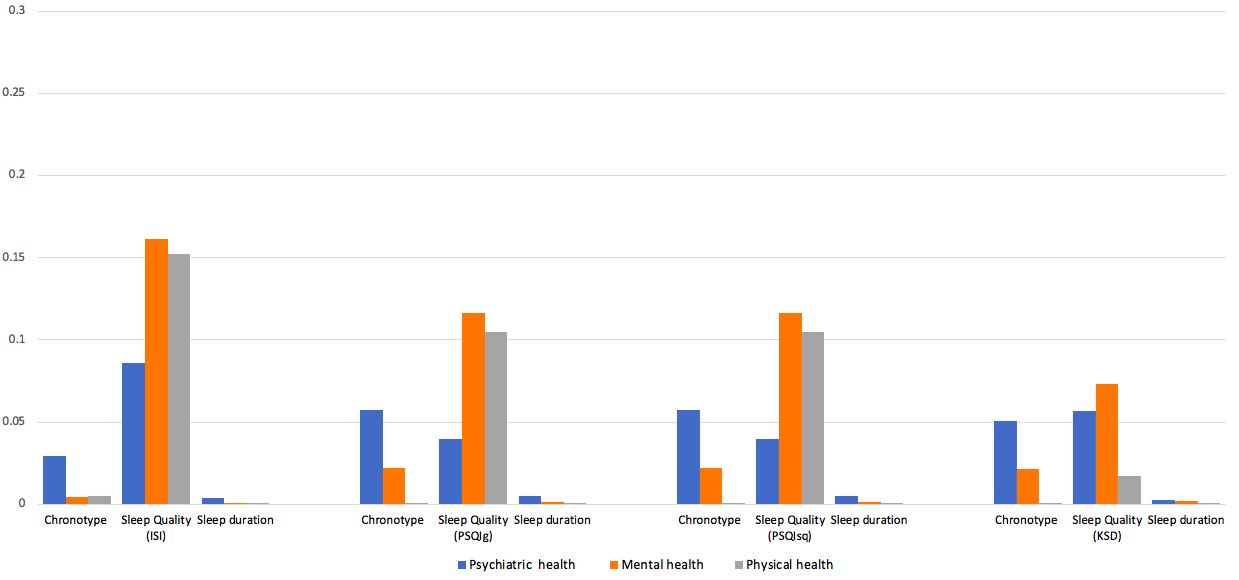
**

**Women**

**
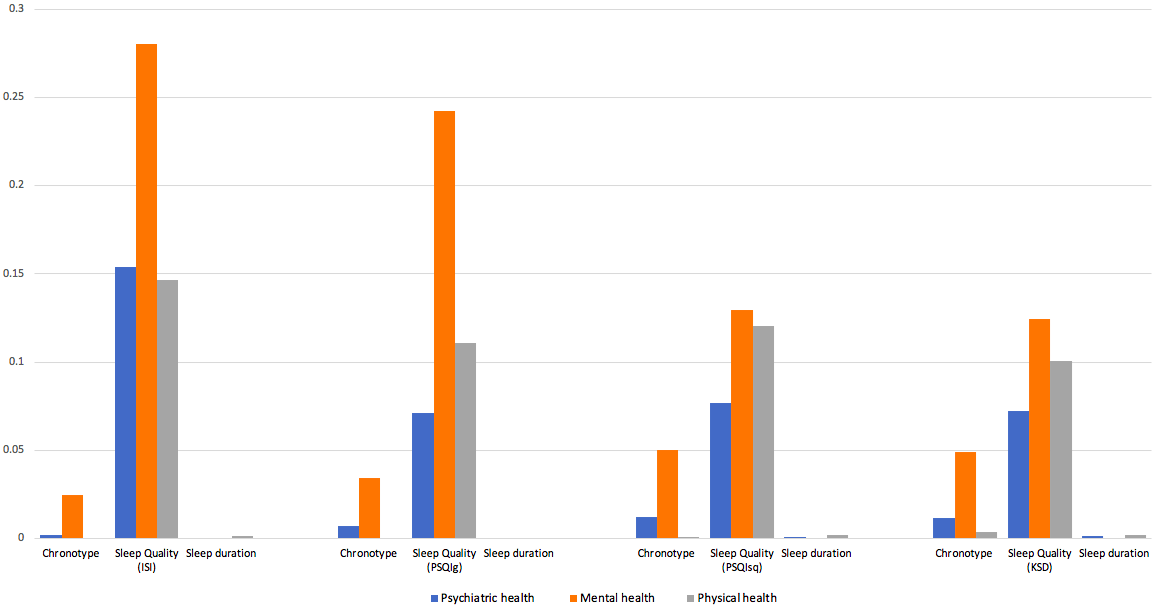
**

A visual summary of the local effect sizes of chronotype, sleep duration and different sleep quality measures on the three main health outcomes for each sex group. Chronotype measure is based on total scores of the Morningness-Evenigness Questionnaire (MEQ), sleep duration measure is based on the average sleep period time calculation from the, Munich Chronotype Questionnaire (MCTQ) and sleep quality measures is from the Insomnia Severity Index (ISI), global score and single question on sleep quality from the Pittsburgh Sleep Quality Index (PSQI) and the sleep quality measure from the Karolinska Sleep Diary (KSD).

**REFERENCES**

Akerstedt, T., Hume, K., Minors, D., & Waterhouse, J. (1994). The meaning of good sleep: a longitudinal study of polysomnography and subjective sleep quality. *J Sleep Res, 3*(3), 152-158. doi:10.1111/j.1365-2869.1994.tb00122.x

Allebrandt, K. V., Teder-Laving, M., Akyol, M., Pichler, I., Muller-Myhsok, B., Pramstaller, P., . . . Roenneberg, T. (2010). CLOCK gene variants associate with sleep duration in two independent populations. *Biol Psychiatry, 67*(11), 1040-1047. doi:10.1016/j.biopsych.2009.12.026

Bastien, C. H., Vallieres, A., & Morin, C. M. (2001). Validation of the Insomnia Severity Index as an outcome measure for insomnia research. *Sleep Med, 2*(4), 297-307. doi:10.1016/s1389-9457(00)00065-4

Buysse, D. J., Reynolds, C. F., 3rd, Monk, T. H., Berman, S. R., & Kupfer, D. J. (1989). The Pittsburgh Sleep Quality Index: a new instrument for psychiatric practice and research. *Psychiatry Res, 28*(2), 193-213. doi:10.1016/0165-1781(89)90047-4

Carver, C. S., & White, T. L. (1994). Behavioral inhibition, behavioral activation, and affective responses to impending reward and punishment: The BIS/BAS Scales. *Journal of Personality and Social Psychology, 67*(2), 319-333. doi:10.1037/0022-3514.67.2.319

Devine, E. B., Hakim, Z., & Green, J. (2005). A systematic review of patient-reported outcome instruments measuring sleep dysfunction in adults. *Pharmacoeconomics, 23*(9), 889-912. doi:10.2165/00019053-200523090-00003

Goldberg, D. (1978). *Manual of the general health questionnaire*. Windsor: NFER.

Goldberg, L. R. (1990). An alternative "description of personality": the big-five factor structure. *J Pers Soc Psychol, 59*(6), 1216-1229. doi:10.1037//0022-3514.59.6.1216

Groeger, J. A., Zijlstra, F. R., & Dijk, D. J. (2004). Sleep quantity, sleep difficulties and their perceived consequences in a representative sample of some 2000 British adults. *J Sleep Res, 13*(4), 359-371. doi:10.1111/j.1365-2869.2004.00418.x

Horne, J. A., & Ostberg, O. (1976). A self-assessment questionnaire to determine morningness-eveningness in human circadian rhythms. *Int J Chronobiol, 4*(2), 97-110.

McCrae, R. R., & Costa, P. T., Jr. (1987). Validation of the five-factor model of personality across instruments and observers. *J Pers Soc Psychol, 52*(1), 81-90. doi:10.1037//0022-3514.52.1.81

Roenneberg, T., Wirz-Justice, A., & Merrow, M. (2003). Life between clocks: daily temporal patterns of human chronotypes. *J Biol Rhythms, 18*(1), 80-90. doi:10.1177/0748730402239679

Selya, A. S., Rose, J. S., Dierker, L. C., Hedeker, D., & Mermelstein, R. J. (2012). A Practical Guide to Calculating Cohen's f(2), a Measure of Local Effect Size, from PROC MIXED. *Front Psychol, 3*, 111. doi:10.3389/fpsyg.2012.00111

Taillard, J., Philip, P., Chastang, J. F., & Bioulac, B. (2004). Validation of Horne and Ostberg morningness-eveningness questionnaire in a middle-aged population of French workers. *J Biol Rhythms, 19*(1), 76-86. doi:10.1177/0748730403259849

Tonetti, L. (2007). Validity of the Morningness-Eveningness Questionnaire for Adolescents (MEQ-A). *Sleep and Hypnosis, 9*(2), 47-51.

van Strien, T., Frijters, J. E. R., Bergers, G. P. A., & Defares, P. B. (1986). The Dutch Eating Behavior Questionnaire (DEBQ) for assessment of restrained, emotional, and external eating behavior. *International Journal of Eating Disorders, 5*(2), 295-315. doi:10.1002/1098-108x(198602)5:2<295::aid-eat2260050209>3.0.co;2-t

Ware, J. E., Jr., & Sherbourne, C. D. (1992). The MOS 36-item short-form health survey (SF-36). I. Conceptual framework and item selection. *Med Care, 30*(6), 473-483.

Watson, D., Clark, L. A., & Tellegen, A. (1988). Development and validation of brief measures of positive and negative affect: The PANAS scales. *Journal of Personality and Social Psychology, 54*(6), 1063-1070. doi:10.1037/0022-3514.54.6.1063
